# Gene Expression and Autoantibody Analysis Revealing Distinct Ancestry‐Specific Profiles Associated With Response to Rituximab in Refractory Systemic Lupus Erythematosus

**DOI:** 10.1002/art.42404

**Published:** 2023-03-22

**Authors:** Lucy M. Carter, Adewonuola Alase, Zoe Wigston, Antonios Psarras, Agata Burska, Emily Sutton, Md Yuzaiful Md Yusof, John A. Reynolds, The MASTERPLANS Consortium, Neil McHugh, Paul Emery, Miriam Wittmann, Ian N. Bruce, Edward M. Vital

**Affiliations:** ^1^ Leeds Institute of Rheumatic and Musculoskeletal Medicine, University of Leeds, and NIHR Leeds Biomedical Research Centre, Leeds Teaching Hospitals NHS Trust Leeds UK; ^2^ Leeds Institute of Rheumatic and Musculoskeletal Medicine, University of Leeds Leeds UK; ^3^ Centre for Epidemiology Versus Arthritis, Division of Musculoskeletal and Dermatological Sciences University of Manchester Manchester UK; ^4^ Institute of Inflammation and Ageing, University of Birmingham, and Sandwell and West Birmingham NHS Trust Birmingham UK; ^5^ Department of Pharmacy and Pharmacology University of Bath, Claverton Bath UK; ^6^ Department of Dermatology University Medical Centre, Johannes Gutenberg‐University Mainz Germany

## Abstract

**Objective:**

Gene expression profiles are associated with the clinical heterogeneity of systemic lupus erythematosus (SLE) but are not well studied as biomarkers for therapy. We studied gene expression and response to rituximab in a multiethnic UK cohort who were refractory to standard therapy.

**Methods:**

We evaluated baseline expression levels of transcripts known to associate with clinical features of SLE using a 96‐probe TaqMan array and whole blood samples from 213 patients with active SLE who had been prospectively enrolled in the British Isles Lupus Assessment Group (BILAG) Biologics Register. We measured autoantibodies using immunoprecipitation and enzyme‐linked immunosorbent assays. We determined responses to first‐cycle rituximab at 6 months from treatment start in 110 SLE patients by assessing BILAG 2004 disease activity.

**Results:**

Interferon gene expression scores were lower in patients of European ancestry than in all other ancestry groups. The relationship between blood interferon gene expression scores and scores annotated to plasmablasts, neutrophils, myeloid lineage, inflammation, and erythropoiesis differed between patients of European and non‐European ancestries. Hierarchical clustering revealed 3 distinct non‐European ancestry patient subsets with stratified responses to rituximab that were not explained by sociodemographic and clinical variables, with responses lowest in an interferon‐low, neutrophil‐high cluster and highest in a cluster with high expression levels across all signatures (*P* < 0.001). Clusters in European ancestry patients did not predict response to rituximab but segregated patients by global disease activity and renal involvement. In both ancestral groups, interferon‐high clusters were associated with U1 RNP/Sm antibodies.

**Conclusion:**

Ancestry appears central to the immunologic and clinical heterogeneity in SLE. These results suggest that ancestry, disease activity, and transcriptional signatures could each assist in predicting the effectiveness of B cell depletion therapies.

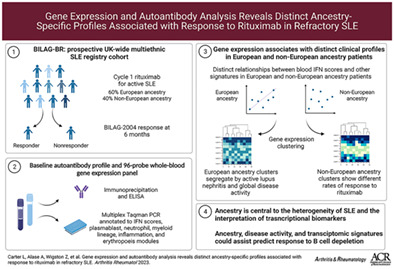

## INTRODUCTION

Systemic lupus erythematosus (SLE) is a complex multisystem disease in which immune dysregulation culminates in autoantibodies to nuclear antigens, immune complex deposition, complement activation, and tissue injury (1). The underlying immunopathologic diversity contributes to variabilities in disease severity, response to therapy, and clinical outcomes, which are yet to be completely understood. Heterogeneity between ancestral groups appears particularly important, although minority ancestral groups remain underrepresented in most clinical studies (2). Non‐European ancestry populations frequently show higher prevalence and younger SLE onset than populations of European ancestry (3), as well as greater renal involvement and damage accrual (4, 5). Improved stratification between and within ancestral groups could therefore be a crucial strategy to improve treatment selection and achieve greater parity in clinical outcomes.

B cell depletion using the anti‐CD20 monoclonal antibody rituximab is an important therapeutic strategy in patients with refractory SLE (6). Despite its widespread use, initial clinical trials did not meet their primary end points, and patient response can vary markedly (7, 8). Patients of African ancestry showed greater response to rituximab in 1 major trial (9) but appeared less responsive to B cell–directed therapy with the BAFF‐neutralizing monoclonal antibody agent belimumab (10). Differential efficacy across other minority ancestral groups, such as patients from subcontinental Asia, is less well characterized, and it is not always clear to what extent differences in outcomes are influenced by geographic and social factors influencing access to health care.

Gene expression profiles show the potential to assist SLE stratification. The blood transcriptome of SLE has been comprehensively evaluated by microarray (11, 12), permitting the assembly of coexpressed transcripts into functionally annotated modules that distinguish by disease activity, autoantibody status (11), renal involvement (12, 13), and cutaneous manifestations (14). Well‐characterized autoantibody clusters to RNA‐binding proteins have been associated with certain clinical phenotypes, interferon (IFN) signatures, and less favorable responses to B cell depletion (15, 16, 17). We have previously validated 2 continuous IFN gene expression scores, IFN‐Score‐A and IFN‐Score‐B, which were derived from factor analysis of IFN‐annotated modules and which yielded stronger clinical associations than a more global IFN signature (18, 19). Ancestral background significantly influences IFN signatures (20) and other transcriptional profiles in SLE (21). Although B cell dynamics after rituximab therapy can predict subsequent outcomes, pretreatment biomarkers that predict response are lacking (22, 23, 24). Gene expression profiles associated with response to rituximab in SLE have not yet been evaluated, and ancestry‐specific effects have not yet been explored.

In the present study, we examined the relationship between ancestry, whole‐blood gene expression signatures, autoantibody status, and response to first‐cycle rituximab therapy in a multiethnic UK cohort of patients with SLE who were disease refractory to standard therapy. This work is part of the MASTERPLANS consortium, which aims to identify predictors of response and stratified approaches to treatment of SLE.

## PATIENTS AND METHODS

### Patients

The British Isles Lupus Assessment Group (BILAG) Biologics Register is a prospective UK‐wide registry evaluating the safety and efficacy of biologics in SLE. Study approval was obtained from the North West–Greater Manchester West National Research Ethics Service Committee (Research Ethics Committee no. 09/H1014/64) and the UK Health Research Authority (Integrated Research Application System no. 24407). Eligibility for rituximab in England requires cyclophosphamide and/or mycophenolate mofetil treatment failure, active SLE (at least 1 BILAG grade A manifestation and/or 2 BILAG grade B manifestations, or Systemic Lupus Erythematosus Disease Activity Index 2000 [SLEDAI‐2K] score ≥ 6) (25), or unacceptably high dose of glucocorticoids to control disease (26, 27). Comprehensive clinical and demographic data, including clinical hematology and immunology obtained through local diagnostic laboratories, were captured prospectively. Patient self‐identified ancestry was recorded according to the UK Office of National Statistics 2011 census categories. Socioeconomic deprivation was measured by the 2019 Index of Multiple Deprivation (IMD) rank of the statistical geography of postal address on enrolment (Supplementary Methods, available on the *Arthritis & Rheumatology* website at https://onlinelibrary.wiley.com/doi/10.1002/art.42404) (https://www.gov.uk/government/statistics/english-indices-of-deprivation-2019). The primary rituximab response criterion was evaluated in 110 patients with either ≥1 BILAG grade A or ≥ 2 BILAG grade B manifestations at baseline (Supplementary Figure 1, available at https://onlinelibrary.wiley.com/doi/10.1002/art.42404).

### Clinical outcomes

Disease activity was assessed using the BILAG 2004 index (28, 29). Response in SLE patients was defined as improvement in all BILAG A scores and no more than 1 persisting BILAG grade B score at 6 months after treatment, with no new BILAG grade A/B flares (22).

### Whole blood gene expression analysis

We evaluated gene expression levels using whole blood samples obtained in Tempus tubes from 213 SLE patients before rituximab treatment, with blinding of participant's clinical status. For gene expression analysis, we used a customized 96‐probe TaqMan array as previously described (18). Ct values were normalized to levels of the reference gene peptidylprolyl isomerase A (PPIA), and ΔCt was reflected such that higher values indicated greater gene expression.

### Gene selection and gene expression scores

IFN‐annotated transcripts comprised 2 validated continuous expression scores for IFN‐stimulated genes (ISGs) (18) and 7 additional well‐characterized ISGs. IFN‐Score‐A includes transcripts most frequently reported in global type I IFN signatures. IFN‐Score‐B includes additional ISGs that may be dynamically responsive to multiple IFN subtypes. Genes annotated to plasmablasts (n = 4; M4.11, M7.7), neutrophils (n = 15; M5.15), myeloid lineage (n = 17; M3.2, M5.7), inflammation (n = 13; M4.2), and erythropoiesis (n = 11; M2.3, M3.1) were selected from previously described modules based on known molecular function and attributes (11). Supplementary Table 1 shows a complete listing of transcripts and corresponding TaqMan IDs (available on the *Arthritis & Rheumatology* website at https://onlinelibrary.wiley.com/doi/10.1002/art.42404). Gene expression scores for each annotation were represented by the median reflected ΔCt results of the relevant transcripts.

### Immunoprecipitation and enzyme‐linked immunosorbent assays

Autoantibody analysis was performed at a specialist autoimmune serology laboratory at the University of Bath for a subset of patients using serum contemporaneous to gene expression. Serotyping for Ro 60, La, and RNP/Sm was performed by radiolabeled protein immunoprecipitation assays as previously described (30, 31). Anti–SSA 52 IgG (Abnova), anticardiolipin IgG III, and anti–double‐stranded DNA (anti‐dsDNA) IgG (both Quanta Lite; Inova Diagnostics) were evaluated by enzyme‐linked immunosorbent assays (Supplementary Methods, available on the *Arthritis & Rheumatology* website at https://onlinelibrary.wiley.com/doi/10.1002/art.42404).

### Statistical analysis

We used R version 4.1.1 and R Studio version 1.3.1093 for statistical analyses. Polymerase chain reaction Ct values falling below the prespecified minimum signal intensity were imputed with the nondetects package (32). For hierarchical clustering, the complete linkage method in package hclust was used. For data visualization, we used ggplot2, heatmaps, Corrplot, and ComplexUpset (33). Correlation was assessed by Spearman's correlation coefficient. Normally distributed continuous variables were compared by *t*‐tests or analysis of variance and Tukey's honestly significant difference post hoc test. The Kruskal‐Wallis test and the Dunn's post hoc test were used for comparisons of nonparametric variables. Categorical variables were compared by chi‐square test. *P* values less than or equal to 0.05 were considered significant.

## RESULTS

### Study population

Our study cohort included 213 patients with SLE enrolled in the BILAG Biologics Register who had pretreatment whole blood available for gene expression analysis. Of 213 patients, 162 (76%) were enrolled upon starting rituximab (Supplementary Figure 1, available on the *Arthritis & Rheumatology* website at https://onlinelibrary.wiley.com/doi/10.1002/art.42404). Of 213 patients, 128 (60%) were of White European ancestry, specifically White British (55%) or Irish (5%). Minority ancestral groups included 13% with African ancestry (n = 27 of 213), 13% with subcontinental Asian ancestry (n = 27 of 213), and 5% with Chinese and Other Asian heritage (n = 11 of 213). Other ancestral backgrounds, including mixed ethnicity, accounted for the remaining 9% of SLE patients in the cohort. Compared with patients of European ancestry (n = 128), patients of non‐European ancestry collectively (n = 85) were significantly younger (37 versus 43 years; *t*‐test = −3.4, *P* = 0.001), had lower prevalence of cigarette smoking (19% versus 44%; chi‐square test = 10.4, *P* = 0.001), and resided in areas of significantly higher overall relative deprivation (IMD rank 17,526 versus 11,311; *t*‐test = 3.1, *P* = 0.002) and higher relative deprivation in 6 of 7 composite IMD domains (Supplementary Figure 2, available on the *Arthritis & Rheumatology* website at https://onlinelibrary.wiley.com/doi/10.1002/art.42404).

In our study cohort, SLE patients of non‐European ancestry had higher rates of hypocomplementemia (57% versus 43.0%; chi‐square test = 4.0, *P* = 0.045), higher total IgG levels (16.2 gm/liter versus 10.9 gm/liter; *t*‐test = 4.8, *P* < 0.000), and higher seropositivity for U1 RNP/Sm antibodies (50% versus 12%; chi‐square test = 28.6, *P* < 0.000), Ro 60 antibodies (45% versus 29%; chi‐square test = 4.2, *P* = 0.040), and anti‐dsDNA antibodies (68% versus 46%; chi‐square test = 7.3, *P* = 0.006). We observed no substantive differences in disease activity (SLEDAI‐2K and numerical BILAG), type of therapy at registration, or type of concomitant SLE therapies. Full clinical and demographic characteristics of the SLE study cohort are summarized in Supplementary Table 2 (available at https://onlinelibrary.wiley.com/doi/10.1002/art.42404).

### Varied relationships between annotated gene expression scores according to ancestry in SLE patients

**Figure 1:**
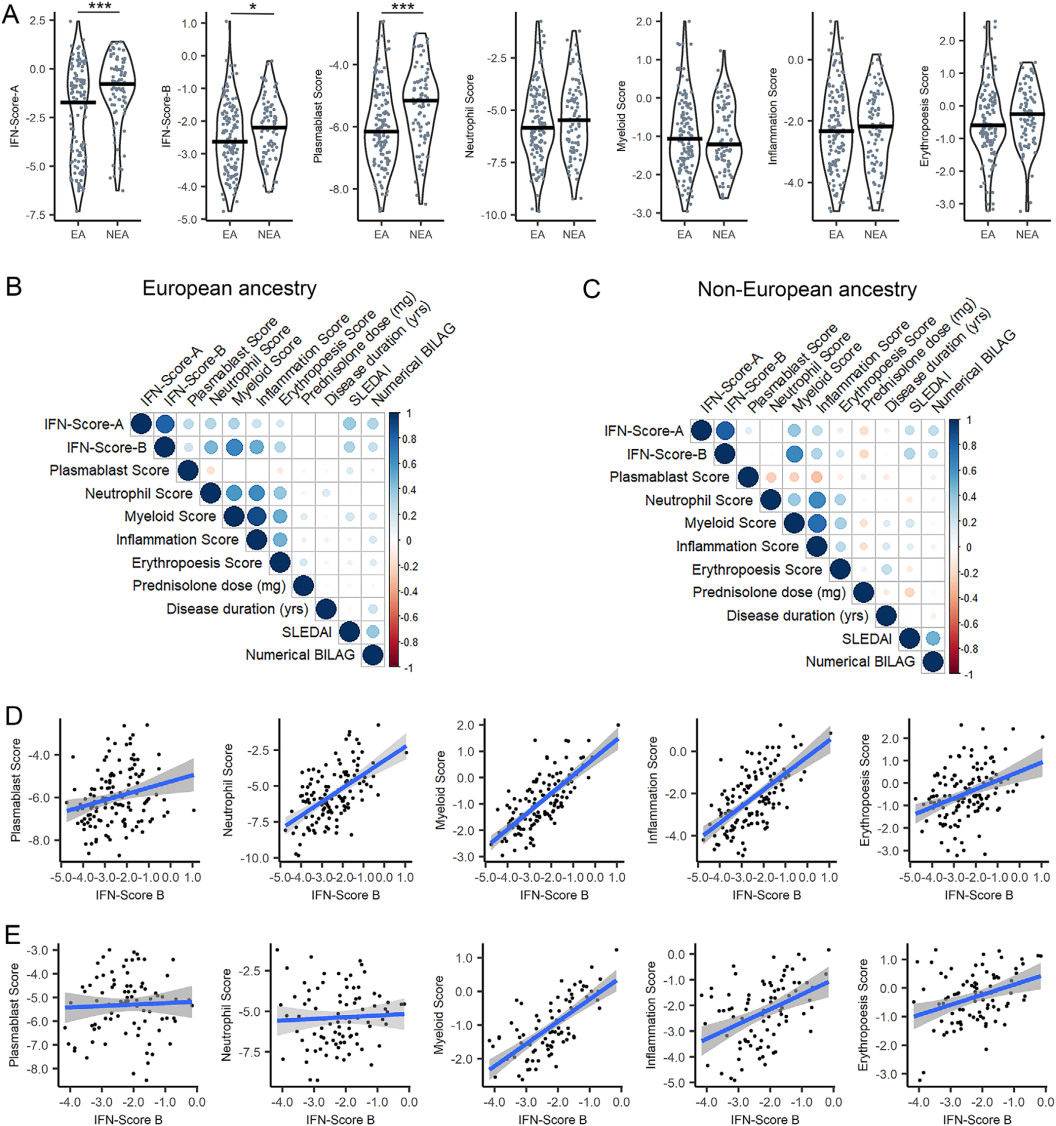
Varied expression levels and interrelationships among annotated gene expression scores by ancestry in patients with systemic lupus erythematosus (SLE). **A**, Violin plots with overlay jitter points showing distribution of IFN‐Score‐A and IFN‐Score‐B (2 validated continuous expression scores for interferon [IFN]‐stimulated genes) and gene expression scores annotated to plasmablasts, neutrophils, myeloid lineage, and inflammation in SLE patients of European ancestry (EA; n = 128) and non‐European ancestry (NEA; n = 85). Lines inside the plots represent the median. * = *P* < 0.05; *** = *P* < 0.001. **B** and **C**, Matrix correlograms showing the strength (as indicated by circle size and color intensity) of positive (blue) or negative (red) Spearman's correlation coefficients for associations between annotated gene expression scores and selected clinical variables for SLE patients of European (**B**) and non‐European (**C**) ancestry. **D** and **E**, Scatterplots showing associations between gene expression scores annotated to plasmablasts, neutrophils, myeloid lineage, inflammation, and erythropoiesis and IFN pathway activation, as measured by IFN‐Score‐B, in patients of European (**D**) and non‐European (**E**) ancestry. Lines show regression, and shaded areas show SE. All gene expression scores are shown as ΔCt from reference gene peptidylprolyl isomerase A (PPIA) reflected across zero, such that higher values indicate higher expression levels. SLEDAI = Systemic Lupus Erythematosus Disease Activity Index; BILAG = British Isles Lupus Assessment Group.

IFN‐Score‐A, IFN‐Score‐B, and gene expression scores annotated to plasmablasts, neutrophils, myeloid lineage, inflammation, and erythropoiesis showed distinct profiles associated with SLE patient ancestry. Consistent with previous literature, the IFN signature expression, measured by IFN‐Score‐A, showed marked separation between European and non‐European UK ancestries (Supplementary Figure 3, available on the *Arthritis & Rheumatology* website at https://onlinelibrary.wiley.com/doi/10.1002/art.42404). SLE patients of European ancestry showed lower median expression of IFN‐Score‐A (−1.72 versus −0.77; Kruskal‐Wallis test = 3827, *P* = 0.0002), with a bimodal distribution that was not apparent among SLE patients of non‐European ancestry (Figure 1A and Supplementary Figure 3). SLE patients of European ancestry also displayed lower IFN‐Score‐B (−2.62 versus −2.19; *t*‐test = 2.13, *P* = 0.034) and plasmablast‐annotated gene expression scores (−6.15 versus −5.15; *t*‐test = 3.73, *P* = 0.0002) (Figure 1A).

Among patients of European ancestry in our SLE cohort (n = 128), gene expression scores across all annotations were closely aligned with IFN pathway activation. IFN‐Score‐B, which comprises ISGs sensitive to multiple IFN subtypes, was significantly and positively correlated with gene expression scores annotated to plasmablasts (R^2^ = 0.265, *P* = 0.002), neutrophils (R^2^ = 0.530, *P* < 0.000), myeloid lineage (R^2^ = 0.714, *P* < 0.000), inflammation (R^2^ = 0.598, *P* < 0.000), and erythropoiesis (R^2^ = 0.437, *P* < 0.000) (Figures 1B and D).

In contrast, among SLE patients of non‐European ancestry (n = 85), plasmablast and neutrophil gene expression scores were completely dissociated from IFN status. We observed no significant correlation between IFN‐Score‐B and gene expression scores annotated to plasmablasts (R^2^ = 0.001, *P* = 0.990) or neutrophils (R^2^ = 0.109, *P* = 0.318) (Figures 1C and E). However, a strong positive correlation was retained in non‐European ancestry patients between IFN‐Score‐B and gene expression scores annotated to myeloid lineage (R^2^ = 0.716, *P* < 0.000), inflammation (R^2^ = 0.445, *P* < 0.000), and erythropoiesis (R^2^ = 0.296, *P* = 0.006) (Figures 1C and E). Similar relationships were observed with IFN‐Score‐A, although the strength of the correlation, when present, was weaker than for IFN‐Score‐B (Figures 1B and C and Supplementary Figure 4, available on the *Arthritis & Rheumatology* website at https://onlinelibrary.wiley.com/doi/10.1002/art.42404). The same pattern was consistent across SLE patients of African ancestry and subcontinental Asia when evaluated discretely (Supplementary Figure 5, available at https://onlinelibrary.wiley.com/doi/10.1002/art.42404).

Among SLE patients of European ancestry, IFN‐Score‐A and IFN‐Score‐B were both positively correlated with overall disease activity, with a stronger relationship for SLEDAI‐2K (for IFN‐Score‐A, R^2^ = 0.366, *P* < 0.000; for IFN‐Score‐B, R^2^ = 0.333, *P* < 0.000) than numerical BILAG (for IFN‐Score‐A, R^2^ = 0.282, *P* = 0.002; for IFN‐Score‐B, R^2^ = 0.224, *P* = 0.013). In contrast, among SLE patients of non‐European ancestry, IFN status was not related to overall disease activity, with no significant correlation shown between either IFN‐Score‐A and IFN‐Score‐B and SLEDAI‐2K (for IFN‐Score‐A, R^2^ = 0.159, *P* = 0.156; for IFN‐Score‐B, R^2^ = 0.194, *P* = 0.083) or numerical BILAG (for IFN‐Score‐A, R^2^ = 0.174, *P* = 0.128; for IFN‐Score‐B, R^2^ = 0.133, *P* = 0.247).

Several transcriptomic features were common to both ancestral groups. Specifically, there was significant positive correlation between gene expression scores annotated to neutrophils, myeloid lineage, and inflammation (Figures 1B and C) in SLE patients of European ancestry and of non‐European ancestry. Similarly, in both ancestral groups, we observed a positive correlation between IFN‐Score‐A and gene expression scores annotated to myeloid lineage, inflammation, and erythropoiesis (Supplementary Figure 4, available at https://onlinelibrary.wiley.com/doi/10.1002/art.42404). Neither IFN‐Score‐A nor IFN‐Score‐B showed a significant relationship with disease duration or current glucocorticoid doses (Figures 1B and C).

### Ancestry‐restricted relationships between gene expression scores and organ domain involvement

**Figure 2:**
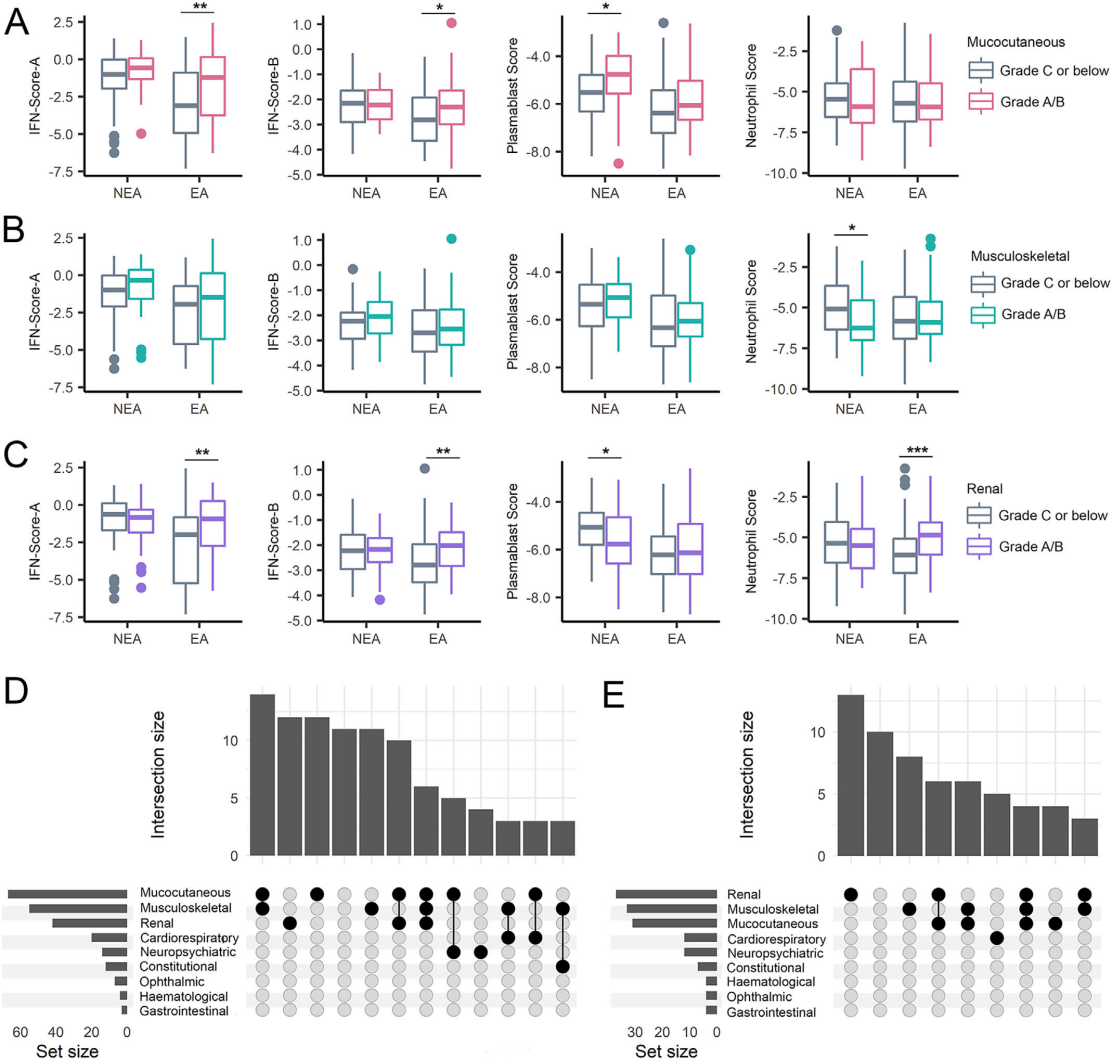
Gene expression scores differentially associated with BILAG 2004 domain activity in SLE patients of European and non‐European ancestry. **A–C**, Boxplots showing IFN‐Score‐A, IFN‐Score‐B, and gene expression scores annotated to plasmablasts and neutrophils in SLE patients of European ancestry (EA) and non‐European ancestry (NEA). Associations between gene expression scores and BILAG 2004 grade A/B disease activity are shown for the mucocutaneous (**A**), musculoskeletal (**B**), and renal (**C**) domains compared with patients with lower (grade C or below) domain activity. Whiskers represent the highest and lowest value, and boxes represents the upper and lower interquartile range. Lines inside the boxes represent the median. Solid circles indicate outliers. * = *P* < 0.05; ** = *P* < 0.01; *** = *P* < 0.001. **D** and **E**, Upset plots for SLE patients of European ancestry (**D**) and non‐European ancestry (**E**) showing frequency of BILAG 2004 grade A/B activity (bar chart, intersection size) according to BILAG 2004 domain coinvolvement (dot‐connectivity plot, group). Upset plot horizontal bar chart (set size) shows the frequency of grade A/B activity by each individual BILAG 2004 domain. See Figure 1 for definitions.

The relationship between gene expression scores and active BILAG 2004 grade A/B disease (compared with grade C or lower) varied between the European and non‐European ancestral groups in our SLE patient cohort. Among SLE patients of European ancestry, active mucocutaneous and renal disease were associated with a significantly higher mean IFN‐Score‐A (mucocutaneous domain 1.793 versus −2.930; *t*‐test = −2.65, *P* = 0.008; and renal domain −1.375 versus −2.794; *t*‐test = −3.45, *P* = 0.001) and IFN‐Score‐B (mucocutaneous domain −2.308 versus −2.708; *t*‐test = −2.08, *P* = 0.040; and renal domain −0.196 versus −0.938; *t*‐test = −3.0, *P* = 0.003). Active renal disease was strongly associated with a higher neutrophil score (−4.899 versus −5.994, *t*‐test = −3.5, *P* = 0.000) in SLE patients of European ancestry. Active musculoskeletal disease was not distinguished by any score among SLE patients of European ancestry (Figure 2B).

In contrast, among SLE patients of non‐European ancestry, IFN scores were not associated with active disease in any of the examined organ domains (Figure 2). However, active mucocutaneous disease in SLE patients of non‐European ancestry was associated with a higher mean gene expression score annotated to plasmablasts (−4.940 versus −5.581, *t*‐test = −2.2, *P* = 0.033) (Figure 2A). Active renal disease among SLE patients of non‐European ancestry was conversely associated with a lower plasmablast gene expression score (−5.668 versus −5.031, *t*‐test = 2.5, *P* = 0.028) (Figure 2C). Unlike that shown among SLE patients of European ancestry, the neutrophil gene expression score did not distinguish between active and inactive renal involvement (Figure 2C), but active musculoskeletal disease was associated with a lower neutrophil score (−5.892 versus −4.958, *t*‐test = 2.1, *P* = 0.018) (Figure 2B).

The co‐occurrence of BILAG 2004 grade A/B involvement across organ systems is shown in Figure 2. Among SLE patients of European ancestry, mucocutaneous disease was most prevalent overall. The most frequent patterns of organ involvement were co‐occurring mucocutaneous and musculoskeletal disease followed by single‐organ renal disease and single‐domain mucocutaneous disease (Figure 2D). Among SLE patients of non‐European ancestry, renal disease was most prevalent overall, and single‐organ renal disease was the most frequent pattern of involvement, followed by single‐domain musculoskeletal disease and concurrent active renal and mucocutaneous activity (Figure 2E).

### Distinct disease profiles in SLE patients of European and non‐European ancestry identified by transcript‐level clustering

Unsupervised hierarchical clustering of gene expression levels across the 94 individual genes was performed for SLE patients with European ancestry and non‐European ancestry who were undergoing first‐cycle rituximab treatment. Three patient clusters were each apparent among SLE patients with European ancestry and non‐European ancestry; however, we also observed disease characteristics associated with transcriptional clusters that varied by ancestry.

#### Non‐European ancestry clusters among SLE patients

**Figure 3:**
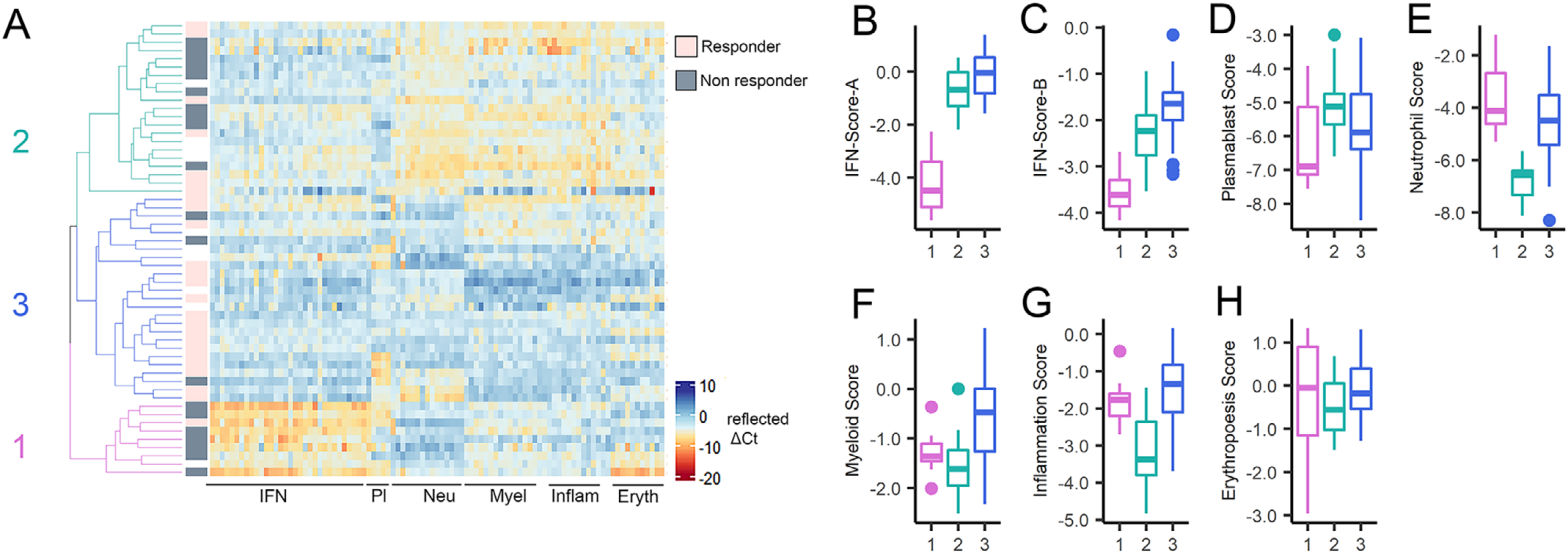
Transcript‐level clustering in SLE patients of non‐European ancestry by differential responses to rituximab. **A**, Heatmap showing expression (reflected as ∆Ct) of 94 transcripts organized by module annotation for SLE patients of non‐European ancestry (n = 55) commencing at cycle 1 of rituximab treatment for active SLE within the BILAG Biologics Register. BILAG 2004 responses to rituximab are identified as responder (rose), nonresponder (gray), or undetermined (white). Dendrogram shows by color the 3 clusters identified by unsupervised hierarchical clustering at the transcript level. Heatmap is centered and scaled by column (transcript). Pl = platelets; Neu = neutrophils; Myel = myeloid lineage; Inflam = inflammation; Eryth = erythropoiesis. **B–H**, Boxplots showing significant differences in gene expression scores annotated to IFN‐Score‐A (**B**), IFN‐Score‐B (**C**), plasmablasts (**D**), neutrophils (**E**), myeloid lineage (**F**), inflammation (**G**), and erythropoiesis (**H**) transcripts according to patient clusters derived from dendrogram in **A**. Whiskers represent the highest and lowest value, and boxes represents the upper and lower interquartile range. Lines inside the boxes represent the median. Solid circles indicate outliers. Cluster 1 (NEA‐1; pale violet red) had low IFN and high neutrophil/myeloid lineage/inflammation scores. Cluster 2 (NEA‐2; sea green) had high IFN and low neutrophil/myeloid lineage/inflammation scores. Cluster 3 (NEA‐3; royal blue) had high expression scores across all annotations. Erythropoiesis‐annotated expression did not differ between clusters. Responders to rituximab had highest prevalence in cluster 3, lowest prevalence in cluster 1, and intermediate prevalence in cluster 2. See Figure 1 for other definitions.

Among SLE patients with non‐European ancestry, 3 clusters were observed (Figure 3). These were termed NEA‐1 (non‐European ancestry with low IFN, high neutrophil/myeloid lineage/inflammation scores), NEA‐2 (non‐European ancestry with high IFN, low neutrophil/myeloid lineage/inflammation scores), and NEA‐3 (non‐European ancestry with high scores for all signatures). Gene expression scores annotated to plasmablasts and erythropoiesis were similar in all clusters. Age, disease duration, and antimalarial use did not significantly differ between clusters. Moreover, we observed no significant differences in baseline disease activity (SLEDAI‐2K and numerical BILAG) or BILAG 2004 organ domain involvement between the 3 clusters (Table 1).

**Table 1:** Clusters derived from gene expression profiles of SLE patients of non‐European ancestry enrolled in the BILAG Biologics Register who were commencing rituximab treatment*

Clinical characteristic	Non‐European ancestry cluster			*P* value
	NEA‐1	NEA‐2	NEA‐3	
	(n = 9)	(n = 21)	(n = 25)	
Ancestry, no. (% of ancestry group)				
African	1 (6)	11 (61)	6 (33)	0.029
South Asian	5 (31)	5 (31)	6 (38)	–
Other Asian (including Chinese)	0 (0)	0 (0)	8 (100)	–
Other (including mixed ancestry)	3 (24)	5 (38)	5 (38)	–
Female patient	8 (89)	17 (81)	22 (88)	0.756
Age, median (IQR) years	45 (32–50)	38 (27–47)	32 (22–39)	0.345
Disease duration, mean (95% CI) years	9 (8–21)	12 (7–16)	13 (8–20)	0.498
Current smoker	4 (44)	2/11 (18)	1/19	0.104
IMD, median (IQR) rank	7,964 (4,972–23,459)	7,126 (2,148–13,019)	16,112 (7,624–23,027)	0.079
BILAG A or B score				
Constitutional	0 (0)	2	1 (4)	–
Mucocutaneous	2 (22)	9 (43)	9 (36)	0.559
Neuropsychiatric	0 (0)	4 (19)	2 (8)	–
Musculoskeletal	2 (22)	9 (43)	8 (32)	0.517
Cardiorespiratory	2 (22)	3 (14)	4 (16)	–
Gastroenterology	0 (0)	1 (4)	3 (12)	–
Ophthalmic	0 (0)	1 (4)	1 (4)	–
Renal	6 (67)	10 (48)	11 (44)	–
Hematology	1 (11)	2 (2)	1 (4)	0.499
BILAG numerical score, median (IQR)	15 (13–20)	21 (13–29)	14 (13–21)	0.381
SLEDAI score, median (IQR)	8 (4–12)	8 (5–14)	8 (4–12)	0.674
SLICC damage index, median (IQR)	1 (0–2)	1 (0–2)	0 (0–1)	0.281
Full blood count, median (IQR)				
Hemoglobin, gm/liter	107.5 (100.2–119.2)	124.5 (103.8–138.2)	114.0 (102.0–116.0)	0.055
White blood cells, × 10^9^/liter	9.6 (9.0–11.5)§	6.1 (3.7–7.3)‡	6.8 (4.1–10.5)	0.007
Neutrophils, × 10^9^/liter	7.6 (6.3–9.2)§	4.5 (2.6–5.6)‡	6.1 (3.1–9.0)	0.011
Lymphocytes, × 10^9^/liter	1.9 (1.2–2.3)	1.0 (0.8–1.7)	0.8 (0.5–1.0)	0.051
Platelets, × 10^9^/liter	256 (131–299)	230 (205–318)	233 (203–286)	0.707
Total IgG, median (IQR) gm/liter	8.0 (6.9–10.6)§	15.1 (12.3–16.7)‡	16.8 (12.5–20.5)‡	0.033
Low C3 or C4	5 (56)	8 (38)	15 (50)	0.255
Concurrent immunosuppressant				
Any agent (MMF, MTX, CNI, AZA)	0 (0)	6 (28)	11 (44)	0.047
Mycophenolate mofetil	0 (0)	4 (19)	9 (36)	0.076
Antimalarial	5 (56)	11 (52)	13 (52)	0.982
Oral glucocorticoid dose, mean (95% CI) mg	20 (5–20)	10 (9.25–15)	10 (6–10)	0.414
Immunoprecipitation and ELISA	(n = 8)	(n = 16)	(n = 19)	
U1 RNP/Sm positive	0 (0)	8 (50)	12 (63)	0.010
Ro 60 positive	4 (50)	4 (25)	9 (47)	0.321
La positive	2 (25)	0 (0)	1 (5)	0.071
Ro 52 ELISA positive	2 (25)	1 (6)	5 (26)	0.276
dsDNA ELISA positive	4 (50)	10 (63)	15 (79)	0.296
Cardiolipin ELISA positive	2 (25)	1 (6)	2 (11)	0.393
Response to rituximab at 6 months	(n = 8)	(n = 17)	(n = 20)	
BILAG responder (complete or partial)	1 (12.5)	7 (41.2)	17 (85.0)	<0.001

* Except where indicated otherwise, values are the number (%) of patients per group. SLE = systemic lupus erythematosus; BILAG = British Isles Lupus Assessment Group; NEA‐1 = low interferon (IFN) score, high neutrophil/myeloid lineage/inflammation scores; NEA‐2 = high IFN score, low neutrophil/myeloid lineage/inflammation scores; NEA‐3 = high scores for all signatures; IQR = interquartile range; 95% CI = 95% confidence interval; IMD = Index of Multiple Deprivation; SLEDAI = Systemic Lupus Erythematosus Disease Activity Index; SLICC = Systemic Lupus International Collaborating Clinics; MMF = mycophenolate mofetil; MTX = methotrexate; CNI = calcineurin inhibitor; AZA = azathioprine; ELISA = enzyme‐linked immunosorbent assay; dsDNA = double‐stranded DNA.

‡ *P* < 0.05 versus NEA‐1 at post hoc analysis.

§ *P* < 0.05 versus NEA‐2 at post hoc analysis.

The NEA‐1 cluster was most clinically and serologically distinct, whereas the NEA‐2 and NEA‐3 clusters were clinically and serologically similar despite markedly different transcriptional profile. Ancestral subgroups did not fully explain these clusters. SLE patients of subcontinental Asian ancestry were equally represented across all 3 clusters. SLE patients of Chinese or other Asian ancestry were exclusively located in the NEA‐3 cluster, although this ancestry group had the fewest number of patients. SLE patients of African ancestry were found in all clusters but were mostly concentrated in the NEA‐2 cluster (chi‐square test = 13.9, *P* = 0.029).

Use of concurrent conventional immunosuppressants was lowest in the NEA‐1 cluster, highest in the NEA‐3 cluster, and intermediate in the NEA‐2 cluster (chi‐square test = 6.08, *P* = 0.047). We observed a trend toward higher glucocorticoid requirement among SLE patients in the NEA‐1 cluster (not statistically significant). Total peripheral white blood cell counts (F = 6.6, *P* = 0.007) and neutrophil counts (F = 5.0, *P* = 0.011) were significantly higher in the NEA‐1 cluster than in the NEA‐2 cluster, with counts for the NEA‐3 cluster falling between these 2 clusters. There was a trend toward increased incidence of anemia in the NEA‐1 cluster (F = 3.1, *P* = 0.055) and a trend toward increased incidence of lymphopenia in the NEA‐3 cluster (F = 3.2, *P* = 0.051). The NEA‐1 cluster was characterized by lower total IgG levels and lower seropositivity for U1 RNP/Sm (chi‐square test = 9.1, *P* = 0.010) compared with the other 2 clusters. There were no significant differences in IgG level or autoantibody status between the NEA‐2 cluster and the NEA‐3 cluster.

#### European ancestry clusters among SLE patients

**Figure 4:**
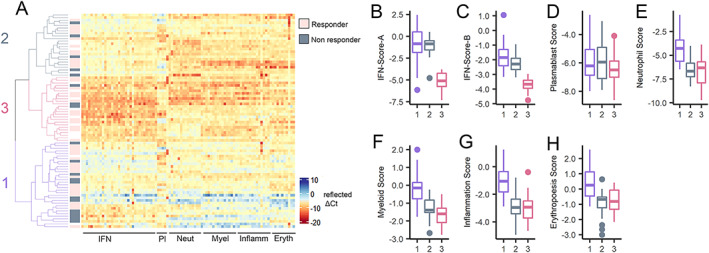
Transcript‐level clustering in SLE patients of European ancestry by disease activity and renal involvement. **A**, Heatmap showing expression (reflected as ∆Ct) of 94 transcripts organized by module annotation for SLE patients of European ancestry (n = 82) commencing at cycle 1 of rituximab treatment for active SLE within the BILAG Biologics Register. BILAG 2004 responses to rituximab are identified as responder (rose), nonresponder (gray), or undetermined (white). Dendrogram shows by color the 3 clusters identified by transcript‐level unsupervised hierarchical clustering of gene expression. Heatmap is centered and scaled by column (transcript). Pl = platelets; Neut = neutrophils; Myel = myeloid lineage; Inflamm = inflammation; Eryth = erythropoiesis. **B–H**, Boxplots showing significant differences in gene expression scores annotated to IFN‐Score‐A (**B**), IFN‐Score‐B (**C**), plasmablast (**D**), neutrophil (**E**), myeloid lineage (**F**), inflammation (**G**), and erythropoiesis (**H**) transcripts according to patient clusters indicated on the dendrogram. Cluster 1 (EA‐1; purple) had high expression across all annotations. Cluster 2 (EA‐2; gray) had high IFN and low neutrophil/myeloid lineage/inflammation/erythropoiesis scores. Cluster 3 (EA‐3; violet red) had low expression across all annotations. Whiskers represent the highest and lowest value, and boxes represents the upper and lower interquartile range. Lines inside the boxes represent the median. Solid circles indicate outliers. See Figure 1 for other definitions.

Among SLE patients of European ancestry, 3 clusters were also evident. These were termed EA‐1 (European ancestry with high scores for all signatures), EA‐2 (European ancestry with high IFN, low neutrophil/myeloid lineage/inflammation/erythropoiesis scores), and EA‐3 (European ancestry with low scores for all signatures) (Figure 4). There were no significant differences in gene expression scores annotated to plasmablasts among the European ancestry clusters (F = 1.4, *P* = 0.238); however, unlike the clusters in SLE patients of non‐European ancestry, we observed significant differences in the erythropoiesis score that paralleled those observed in the gene expression scores annotated to neutrophils, myeloid lineage, and inflammation (Figure 4).

There were no significant differences in age, disease duration, or concurrent use of conventional immunosuppressants and antimalarials among the European ancestry clusters (Table 2). However, in contrast to SLE patients of non‐European ancestry, clusters derived from SLE patients of European ancestry were significantly separated by disease activity as measured by SLEDAI (F = 4.2, *P* = 0.018) and numerical BILAG (F = 4.4, *P* = 0.014) and by BILAG 2004 organ domain involvement (Table 2). We observed that incidence of mucocutaneous and musculoskeletal disease was similarly distributed across all 3 clusters; however, incidence of BILAG 2004 grade A/B renal disease was highly concentrated in the EA‐1 cluster (high scores for all signatures) (chi‐square test = 15.5, *P* < 0.000).

**Table 2:** Clusters derived from gene expression profiles of SLE patients of European ancestry enrolled in the BILAG Biologics Register who were commencing rituximab treatment*

Clinical characteristic	European ancestry cluster			*P* value
	EA‐1	EA‐2	EA‐3	
	(n = 33)	(n = 24)	(n = 25)	
Ancestry				
British	32 (97)	23 (96)	23 (92)	–
Irish	0 (0)	1 (4)	2 (8)	0.402
Other	1 (3)	0 (0)	0 (0)	–
Female patient	29 (88)	24 (100)	23 (92)	0.291
Age, median (IQR) years	41 (33–52)	43 (37–50)	40 (32–46)	0.482
Disease duration, mean (95% CI) years	9 (6–21)	14 (10–17)	9 (7–16)	0.618
Current smoker	10/21 (48)	7/ 20 (35)	10/ 17 (58.5)	0.166
IMD, median (IQR) rank	13,051 (6,083–20,015)	14353 (9,926–22,000)	19,709 (15,340–24,186)	0.090
BILAG A or B score				
Constitutional	5 (15)	2 (8)	3 (12)	–
Mucocutaneous	19 (58)	16 (67)	11 (44)	0.272
Neuropsychiatric	5 (15)	4 (17)	5 (20)	–
Musculoskeletal	17 (52)	12 (50)	8 (32)	0.284
Cardiorespiratory	6 (18)	3 (13)	3 (12)	–
Gastroenterology	0 (0)	2 (8)	1 (4)	–
Ophthalmic	0 (0)	1 (4)	2 (8)	–
Renal	20 (61)	5 (21)	4 (16)	0.000
Hematology	1 (3)	1 (4)	1 (4)	–
BILAG numerical score, mean (95% CI)	22 (16–28)	21 (13–24)	13 (9–20)†	0.014
SLEDAI score, median (IQR)	12 (8–14)	8 (4–11)	6 (2–10)†	0.018
SLICC damage index, median (IQR)	1 (0–2)	1 (0–2)	0 (0–1)	0.381
Full blood count, median (IQR)				
Hemoglobin, gm/liter	121.0 (107.8–131.8)	127.0 (116.0–134.0)	126.0 (114.6–139.0)	0.618
White blood cells, × 10^9^/liter	7.2 (5.2–11.7)	5.8 (5.4–7.6)	6.3 (5.2–7.2)	0.083
Neutrophils, × 10^9^/liter	6.1 (3.6–9.5)‡	4.1 (3.3–6.1)†	3.7 (2.9–5.1)†	0.010
Lymphocytes, × 10^9^/liter	0.9 (0.5–1.3)	1.0 (0.8–1.5)	1.5 (1.1–2.1)†	0.006
Platelets, × 10^9^/liter	279 (228–397)‡	250 (196–278)†	276 (222–355)	0.044
Total IgG, median (IQR) gm/liter	10.8 (8.3–12.8)	11.4 (9.5–14.6)	10.15 (8.0–12.0)	0.224
Low C3 or C4	16 (48)	11 (46)	5 (20)	0.063
Concurrent immunosuppressant				
Any agent (MMF, MTX, CNI, AZA)	13 (39)	10 (42)	13 (52)	0.616
MMF	11 (33)	6 (25)	7 (28)	0.611
Antimalarial	17 (74)	12 (50)	14 (56)	0.907
Oral glucocorticoid dose, mean (95% CI) mg	11 (6–20)	10 (7–12)	11 (8–25)	0.456
Immunoprecipitation and ELISA	(n = 30)	(n = 21)	(n = 22)	
U1 RNP/Sm positive	3 (10)	7 (33)	0 (0)	0.004
Ro 60 positive	8 (27)	7 (33)	5 (23)	0.733
La positive	1 (3)	1 (4)	1 (4)	0.932
Ro 52 ELISA	3 (10)	6 (25)	1 (4)	0.038
dsDNA ELISA	17 (56)	11 (52)	5 (23)	0.054
Cardiolipin ELISA	2 (7)	1 (4)	3 (14)	0.526
Response to rituximab 6 months	(n = 31)	(n = 18)	(n = 16)	
BILAG responder (complete or partial)	19 (61)	13 (72)	13 (81)	0.353

* Except where indicated otherwise, values are the number (%) of patients per group. EA‐1 = high scores for all signatures; EA‐2 = high IFN score, low neutrophil/myeloid lineage/inflammation/erythropoiesis scores; EA‐3 = low scores for all signatures; see Table 1 for other definitions.

† *P* < 0.05 versus EA‐1 at post hoc analysis.

‡ *P* < 0.05 versus EA‐2 at post hoc analysis.

Global disease activity was lowest in the EA‐3 cluster (low scores for all signatures) but was not significantly different from the EA‐1 and EA‐2 clusters despite their differential renal involvement. SLE patients in the EA‐3 cluster, which showed lower disease activity, also had a significantly lower frequency of U1 RNP/Sm seropositivity and a trend toward lower rates of hypocomplementemia and dsDNA antibody positivity (not significant). The clusters differed in mean neutrophil count (F = 4.9, *P* = 0.010) and lymphocyte count (F = 5.5, *P* = 0.006). The EA‐1 cluster (high scores for all signatures), which was characterized by high disease activity and renal involvement, was the only cluster that demonstrated lymphopenia (<1.0 × 10^9^/liter); the EA‐1 cluster also demonstrated higher neutrophil count than the EA‐2 cluster (*P* = 0.050) and the EA‐3 cluster (*P* = 0.013).

Of the transcriptional profiles identified, only the clusters with high scores for all signatures (NEA‐3 and EA‐1) were common to both ancestral groups, although the clinical associations among clusters were distinct. Clusters with high IFN and low neutrophil/myeloid lineage/inflammation scores could be distinguished between European and non‐European ancestries by gene expression scores annotated to erythropoiesis, whereas clusters with low scores for all signatures were unique to SLE patients of European ancestry. Supplementary Table 3 summarizes key cluster characteristics (available on the *Arthritis & Rheumatology* website at https://onlinelibrary.wiley.com/doi/10.1002/art.42404).

### Association between transcriptional profiles in SLE patients of European and non‐European ancestry and response to rituximab

Among SLE patients, 110 patients had evaluable follow‐up data at 6 months after cycle 1 of rituximab treatment (Supplementary Figure 1, available on the *Arthritis & Rheumatology* website at https://onlinelibrary.wiley.com/doi/10.1002/art.42404). Among the 110 SLE patients, 70 (63%) achieved an overall treatment response by BILAG 2004 criteria. Response rate did not significantly differ between SLE patients of European ancestry (45 of 65 [69%]) and SLE patients of non‐European ancestry (25 of 45 [56%]) (chi‐square test = 2.1, *P* = 0.142), and response was not associated with socioeconomic deprivation (*t*‐test = −0.1, *P* = 0.936) (Supplementary Figure 6, available at https://onlinelibrary.wiley.com/doi/10.1002/art.42404). Response was associated with reduction in median oral glucocorticoid dose from 10 mg/day (IQR 5–14) to 5 mg/day (IQR 0–10), and no additional conventional immunosuppressant therapy was registered for any patient in data collected between baseline and 6 months after cycle 1 of rituximab treatment. Supplementary Table 4 summarizes the characteristics of responders and non‐responders in both the European ancestry and non‐European ancestry subsets (available at https://onlinelibrary.wiley.com/doi/10.1002/art.42404). Response by UK census ancestral category is detailed in Supplementary Table 5 (available at https://onlinelibrary.wiley.com/doi/10.1002/art.42404).

Among SLE patients of non‐European ancestry, categorization by transcriptional cluster significantly stratified rituximab response (chi‐square test = 14.5, *P* < 0.001) (Table 1). The NEA‐1 cluster (SLE patients of non‐European ancestry with low IFN and high neutrophil/myeloid lineage/inflammation scores), although comprising the fewest number of patients, was characterized with a distinctly poorer BILAG response to rituximab, with only 12.5% of patients achieving overall response. Both of the non‐European ancestry clusters with high IFN scores (NEA‐2 and NEA‐3) were characterized with a more favorable therapeutic response. The NEA‐3 cluster (high scores for all signatures) showed the highest response rate (17 of 20 [85%] SLE patients of non‐European ancestry). The NEA‐2 cluster (high IFN, low neutrophil/myeloid lineage/inflammation scores), although clinically and serologically similar to NEA‐3, showed a significantly lower rate of overall response (41.2%). This distinctive rituximab response profile between the NEA‐1 cluster and the NEA‐3 cluster was maintained for each composite ancestral group. Heterogeneity in the rituximab response between composite ancestral groups was most pronounced in NEA‐2 (Supplementary Table 6, available on the *Arthritis & Rheumatology* website at https://onlinelibrary.wiley.com/doi/10.1002/art.42404).

In contrast, no significant difference in overall BILAG 2004 response was observed between European ancestry clusters (chi‐square test = 2.1, *P* = 0.353) (Table 2). Although the cluster with low IFN score (NEA‐1) was adversely associated with treatment response among SLE patients of non‐European ancestry, SLE patients of European ancestry in the cluster with low IFN score (EA‐3) had the lowest serologic and clinical disease activity and in fact showed a trend toward a more favorable response.

## DISCUSSION

The stratification of patients that incorporates interacting demographic, clinical, and immunophenotypic features has the potential to assist individualized selection of therapies and to improve overall outcomes of patients with SLE. In our prospective registry evaluation of a multiethnic UK SLE cohort, we demonstrate that transcriptomic signatures differ between ancestral groups and differentially associate with response to rituximab treatment. These results have implications for understanding the pathogenesis of SLE and for improving stratification approaches for evaluating therapeutic interventions.

Epidemiologic studies consistently demonstrate ethnic and geographic differences in the incidence and prevalence of SLE, with disproportionate rates shown among populations of Black and African American, Hispanic, and Asian ancestry compared with populations of White European ancestry (3). SLE patients of non‐European ancestry demonstrate younger onset of disease and greater renal involvement; in addition, SLE patients of African ancestry in particular show higher rates of secondary damage, including atherosclerotic cardiovascular and cerebrovascular disease (4, 34). Furthermore, racial and ethnic disparities in mortality appear only partially attenuated by socioeconomic and geographic factors (35).

Genetic and immunologic studies suggest potential explanations for ancestral differences. So far, >100 SLE susceptibility loci have been identified, with varied roles ranging from nucleic acid processing, IFN pathway involvement, and adaptive immune responses (36). Several genetic risk variants for SLE are not shared between ancestral groups, pointing to diverging heritable immunopathologic mechanisms in different ancestral groups. For example, polymorphism in protein tyrosine phosphatase N22, a negative T cell regulator, is associated with heightened risk of SLE in Hispanic and European populations but not among African ancestry groups (36, 37). Distinct genes and single‐nucleotide polymorphisms are also associated with lupus nephritis risk among SLE patients of different ancestries (38). Notably, genetic variants in IFN regulatory factor (IRF) transcription factors IRF‐5 and IRF‐7 are associated with SLE, and risk haplotypes appear to exert ancestry‐specific effects that are closely linked to serum IFN activity and autoantibody profile (36, 39).

Ancestral differences in DNA methylation associated with several ISGs have also been observed (40). This heterogeneity may help explain why the relationship between IFN pathway activation and other transcriptomic annotations differed between clusters of SLE patients in our study. This observation supports previous analyses. Using a machine‐learning approach, Catalina et al (21) found that ancestry was the dominant influence on whole‐blood gene expression profiles in SLE, above sex, disease characteristics, and therapeutics. Importantly, many modular signatures consistently differed between healthy individuals of different ancestries, with enrichment of granulocyte, inflammasome, and monocyte scores among SLE patients of European ancestry and activated T cell– and B cell–dominant signatures among SLE patients of African ancestry.

To our knowledge, the relationship between gene expression profiles and response to SLE therapies has not been previously investigated. Here, we show that a selected transcriptomic profile associates with organ domain activity and predicts response to rituximab in an ancestry‐specific manner. Although IFN signatures have been previously described as predictors of outcomes in SLE (41), our present data indicate that these are more informative when evaluated in combination with gene expression scores representing other key areas of the SLE transcriptome, as has also recently been explored in juvenile patients with SLE (42). Moreover, apparently similar transcriptional profiles yield distinct disease and prognostic associations for rituximab treatment that are dependent on the ancestral group. The cluster that included high scores for all signatures was associated with a high rituximab response among SLE patients of non‐European ancestry; among SLE patients of European ancestry, however, this cluster was associated with greater renal involvement. In contrast, an SLE patient cluster of European ancestry that included low scores for all signatures lacked an equivalent cluster among SLE patients of non‐European ancestry. Nevertheless, other transcriptomic features were shared between ancestral groups, such as the correlations between IFN‐Score‐A and gene expression signatures annotated to myeloid lineage and inflammation. These profiles could ultimately guide more optimized use of rituximab and may indicate a greater or lesser role for B cells in these immunologic subtypes, but interpretation in an ancestry specific context appears critical.

In stratification studies, it is often unclear whether biomarkers predict response to specific therapies or overall favorable disease natural history. Although we do not have outcome data on other therapies or placebo, eligibility for rituximab in this study did require prior failure to either mycophenolate or cyclophosphamide treatment. Another challenge in stratification studies is understanding the relationship between multiple interacting factors that influence response. Ancestry, autoantibody status, social deprivation, and gene expression all have plausible effects on therapeutic response to rituximab. Indeed, biobehavioral factors linked with sociodemographic conditions may also influence inflammation‐related gene expression (43). Here, we show that stratification of response by the gene expression profile was not influenced by major domains of social deprivation and could distinguish clusters not wholly explained by autoantibody status.

Among SLE patients of non‐European ancestry, we identified a small but very distinctive cluster, NEA‐1 (low IFN, high neutrophil/myeloid lineage/inflammation scores), which demonstrated the poorest response to rituximab. Patients in this cluster showed high disease activity, including significant rates of active renal involvement, and high rates of rituximab treatment failure. Elevated B cells and plasmablast activity, associated with RNP and dsDNA seropositivity, appear more characteristic of SLE patients of non‐European ancestry, particularly those of African ancestry (21). Indeed, in vitro evidence indicates that type I IFN promotes differentiation of B cells toward plasmablasts and plasma cells (44) and their polarization toward proinflammatory phenotypes (45). Expression of BAFF, a key mediator of B cell dynamics, can also be predicted by serum IFN activity and is expressed at a higher level among SLE patients of African American ancestry (46). Thus, stratification of SLE patients of non‐European ancestry by low IFN score and low antibody disease burden (as in cluster NEA‐1) isolates a rituximab‐resistant patient subset, potentially with least B cell–dominant disease. This small cluster comprised a substantial number of SLE patients with subcontinental Asian ancestry, a group that has been sparsely evaluated in the existing literature (47).

The relationship between autoantibodies, ancestry, and IFN status is complex. Consistent with existing literature, we observed that RNP/Sm positivity was enriched in both clusters of SLE patients with high IFN scores (European and non‐European), although with higher prevalence and stronger associations in those of non‐European ancestry (48). Previous studies reveal that the IFN signature among European ancestry patients is also associated with dsDNA seropositivity and may be apparently independent of autoantibodies (49). Our data extend this understanding by showing that gene expression scores outside of the IFN signature refine the clinical associations of the RNP–IFN interaction, particularly with regard to rituximab responsiveness.

Our study has some limitations. Importantly, replication in a validation cohort is still required to verify the transcriptional clusters identified. Additionally, because of the relatively lower numbers of patients with minority ethnic ancestry, our analysis focused on the non‐European ancestries collectively. This work was therefore not able to fully explore heterogeneity within the non‐European ancestry populations and may be underpowered to detect specific features within our less represented groups. Similarly, because distribution of ancestral groups across clusters was not uniform, the influence of individual ancestries to cluster characteristics could not be fully delineated. Further efforts to evaluate ancestral groups discretely are needed. One further consideration is that this work made use of the whole‐blood transcriptomic profile, which has the advantage of relative simplicity for development as a clinically applicable platform but does not permit interrogation of effects driven by differing immune cell population sizes that vary between ancestries. Similarly, this work used a specifically selected subset of transcripts predefined from microarray studies and thus may not capture the effects of other important transcripts that could influence response to rituximab. Because this work did not include a placebo arm, it cannot account for differences in treatment response that are attributable to differences in the natural history of disease. We were also unable to account for differential depth of B cell depletion between groups.

In conclusion, in a UK multiethnic treatment refractory SLE cohort, we observe distinct transcriptomic signatures in SLE that are differentiated by ancestral background and the relationship between IFN pathway activation and other annotated components of the SLE transcriptome. These profiles stratified response to rituximab in an ancestry‐specific manner, and this relationship was not attributable to social deprivation or autoantibody status. Finally, we observed a small subset of patients with active SLE and poor response to rituximab who may have significant unmet needs not addressed by existing SLE therapies. The gene expression profiles employed in this study should be further validated for prediction of response to rituximab. Other studies that aim to stratify lupus trials and to develop biomarkers should consider ancestry, other demographic variables, and patterns of organ involvement alongside overall response. This study adds to a body of work suggesting that there may be subtypes of SLE with less critical roles for B cells as a therapeutic target.

## AUTHOR CONTRIBUTIONS

All authors were involved in drafting the article or revising it critically for important intellectual content, and all authors approved the final version to be published. Dr. Vital had full access to all of the data in the study and takes responsibility for the integrity of the data and the accuracy of the data analysis.

### Study conception and design

Carter, Alase, Reynolds, McHugh, Emery, Wittmann, Bruce, Vital.

### Acquisition of data

Carter, Alase, Wigston, Psarras, Burska, Sutton, Md Yusof, Reynolds, McHugh, Emery, Wittmann, Bruce, Vital.

### Analysis and interpretation of data

Carter, Alase, Wigston, Psarras, Burska, Sutton, Md Yusof, Reynolds, McHugh, Emery, Wittmann, Bruce, Vital.

## Supporting information


Disclosures Form

**Appendix S1:** Supplementary Information

## Data Availability

The data underlying this article are available upon reasonable request from the corresponding author.
