# Supplementary material for: Gene Expression and Autoantibody Analysis Revealing Distinct Ancestry‐Specific Profiles Associated With Response to Rituximab in Refractory Systemic Lupus Erythematosus
Source: Arthritis Rheumatol. 2023 Mar 22;75(5):697–710. doi: 10.1002/art.42404 (PMC10953047; doi:10.1002/art.42404)
Supplement: Supplementary file 2 — Appendix S1: Supplementary Information [file ART-75-697-s002.docx]

**Gene expression and autoantibody analysis reveals distinct ancestry-specific profiles associated with response to rituximab in refractory SLE.**

**Supplementary Material**

Contents:

1. Appendix 1. Supplementary Methods
2. Figure S1. STROBE flow chart of BILAG-BR patients evaluated with gene expression and for response to cycle 1 of rituximab
3. Table S1: Module assignment and Taqman Probe ID for the 94-gene expression panel
4. Figure S2. BILAG-BR patients of non-European ancestry reside in areas of greater relative deprivation.
5. Table S2. Baseline characteristics of BILAG-BR patients of European and non-European ancestry.
6. Figure S3. Interferon and Plasmablast annotated gene expression scores vary by ancestral background.
7. Figure S4. Relationship between IFN-Score-A and other functionally annotated gene expression scores varies by patient ancestry.
8. Figure S5. Commonalities in the relationship between IFN-Score-B and other functionally annotated gene expression scores varies displays are shared between Subcontinental Asian and African ancestry patients.
9. Table S3: Summary of similarities and distinguishing features between gene expression clusters.
10. Figure S6. BILAG response following rituximab therapy is not associated with indices of socioeconomic deprivation.
11. Table S4. Characteristics of BILAG responders and non-responders following cycle 1 rituximab.
12. Table S5. Response to rituximab by UK Census ancestral group
13. Table S6: Differential response to rituximab between non-European ancestry gene expression clusters itemized by patient ancestry.
14. Appendix 2: Contributors to the MASTERPLANS Consortium

## Appendix 1: Supplementary Methods

## Deprivation indices

Index of Multiple Deprivation is the official measure of relative deprivation in England assigned to a standard statistical geography each covering approximately 1,500 residents or 650 households. 2019 IMD combines seven deprivations domains (Income Deprivation, Employment Deprivation, Education, Skills and Training Deprivation, Health Deprivation and Disability, Crime, Barriers to Housing and Services, Living Environment Deprivation). Relative deprivation rank for 2019 IMD and it’s composite domains from 1 (most deprived area) to 32,844 (least deprived area) were derived for patients based on postal address on enrollment to BILAG-Biologics Registry. The 2019 English indices of deprivation datasets, summaries and explanatory material are publicly available from UK Government Ministry of Housing, Communities & Local Government online at:

https://www.gov.uk/government/statistics/english-indices-of-deprivation-2019 [accessed 01 February 2022]

## ELISA

Analysis of all autoantibodies was performed by the specialist autoimmune serology laboratory at the University of Bath. Anti-dsDNA IgG, anti-Ro52 IgG and anti-cardiolipin IgG were measured using the following commercially available kits and according to the manufacturers instructions.

| **Target** | **Kit** | **Specificity** | **Catalogue no.** |
| --- | --- | --- | --- |
| Anti-dsDNA IgG | Inova Diagnostics QUANTA Lite® dsDNA SC ELISA | IgG | 708510 |
| Anti-Ro52 | Abnova SS-A 52 Ab ELISA Kit | IgG | KA1113 |
| Anti-Cardiolipin IgG | Inova Diagnostics QUANTA Lite® ACA IgG III | IgG | 708625 |

Registration therapy

BILAG-BR enrolled patients with available baseline whole blood TEMPUS sample for gene expression analysis

n = 213

MMF

n = 32

Belimumab

n = 19

Rituximab

n = 162

Excluded from clustering analysis

- Insufficient clinical data (n = 25)

EA

n = 16

NEA

n = 16

EA

n = 12

NEA

n = 7

EA

n = 100

NEA

n = 62

Evaluated for

treatment response

at 6 months

n = 110

NEA

n = 45

EA

n = 65

Excluded from response analysis

- Baseline disease activity below pre- specification of primary response criterion (n = 27)

EA

n = 18

NEA

n = 7

NEA

n = 17

NEA

n = 10

## Figure S1. STROBE flow chart of BILAG-BR patients evaluated with gene expression and for response to cycle 1 of rituximab

MMF, mycophenolate mofetil; EA, European ancestry; NEA, Non-European ancestry

| **Table S1. Module assignment and Taqman Probe ID for the 94-gene expression panel** | | | | |
| --- | --- | --- | --- | --- |
| **Gene Symbol** | **Name** | **Module** | **Annotation** | **Probe ID** |
| IFI44L | Interferon induced protein 44 like | 1.2 | IFN Score A | hs00915292_m1 |
| EIF2AK2 | Eukaryotic Translation Initiation Factor 2 Alpha Kinase 2 | 3.4 | ISG | hs00169345_m1 |
| CCL8 | C-C Motif Chemokine Ligand 8 | 3.4 | IFN Score A | hs04187715_m1 |
| ISG15 | Interferon-stimulated gene 15 | 1.2 | IFN Score A | hs00192713_m1 |
| XAF1 | X-linked inhibitor of apoptosis (XIAP)-associated factor 1 | 1.2 | IFN Score A | hs01550142_m1 |
| IFI44 | Interferon Induced Protein 44 | 1.2 | IFN Score A | hs00951349_m1 |
| GBP1 | Guanylate Binding Protein 1 | 3.4 | IFN Score A | hs00977005_m1 |
| IFI27 | Interferon Alpha Inducible Protein 27 | - | IFN Score A | hs01086373_g1 |
| IRF7 | Interferon Regulatory Factor 7 | 3.4 | IFN Score A | hs01014809_g1 |
| CXCL10 | C-X-C motif chemokine ligand 10 | 1.2 | IFN Score A | hs01124251_g1 |
| CEACAM1 | Carcinoembryonic antigen-related (CEA) cell adhesion molecule 1 | 3.4 | IFN Score A | hs00989786_m1 |
| RSAD2 | Radical S-Adenosyl Methionine Domain Containing 2 | 1.2 | IFN Score A | hs00369813_m1 |
| IFIT1 | Interferon Induced Protein With Tetratricopeptide Repeats 1 | 1.2 | IFN Score A | hs01911452_s1 |
| CXCL11 | C-X-C motif chemokine ligand 11 | - | ISG | hs00171138_m1 |
| SIGLEC 1 | Sialic Acid Binding Ig Like Lectin 1 | - | ISG | hs00988063_m1 |
| MX1 | MX Dynamin Like GTPase 1) | 1.2 | ISG | hs00895608_m1 |
| IFI6 | IFI6 interferon alpha inducible protein 6 | - | ISG | hs00242571_m1 |
| HERC5 | HECT And RLD Domain Containing E3 Ubiquitin Protein Ligase 5 | 1.2 | ISG | hs00180943_m1 |
| IFIH1 | Interferon induced with helicase C domain 1 | 3.4 | IFN Score B | hs01070332_m1 |
| CASP1 | Caspase 1 | 5.12 | ISG | hs00354836_m1 |
| SOCS1 | Suppressor of cytokine signaling 1 | 3.4 | IFN Score B | hs00705164_s1 |
| SERPING1 | Serpin Family G Member 1 | 1.2 | IFN Score B | hs00163781_m1 |
| NT5C3B | 5'-Nucleotidase, Cytosolic IIIB | 5.12 | IFN Score B | hs00369454_m1 |
| UNC93B1 | Unc-93 Homolog B1 | 5.12 | IFN Score B | hs00276771_m1 |
| SP100 | SP100 Nuclear Antigen | 5.12 | IFN Score B | hs00162109_m1 |
| TRIM38 | Tripartite Motif Containing 38 | 5.12 | IFN Score B | hs00197164_m1 |
| IFI16 | Interferon Gamma Inducible Protein 16 | 5.12 | IFN Score B | hs00194261_m1 |
| BST2 | Bone Marrow Stromal Cell Antigen 2 | 5.12 | IFN Score B | hs01561315_m1 |
| TAP1 | Transporter 1, ATP Binding Cassette Subfamily B Member | 5.12 | IFN Score B | hs00388675_m1 |
| STAT1 | Signal transducer and activator of transcription 1 | 3.4 | IFN Score B | hs01013996_m1 |
| UBE2L6 | Ubiquitin Conjugating Enzyme E2 L6 | 3.4 | IFN Score B | hs01125548_m1 |
| LAMP3 | Lysosome-associated membrane glycoprotein 3 | 1.2 | IFN Score B | hs00180880_m1 |
| PHF11 | PHD finger protein 11 | 5.12 | IFN Score B | hs00211573_m1 |
| DERL3 | Derlin 3 | 7.7 | Plasmablast | hs00405322_m1 |
| TNFRSF17 | TNF Receptor Superfamily Member 17 | 4.11 | Plasmablast | hs00171292_m1 |
| TXNDC5 | Thioredoxin Domain Containing 5 | 4.11 | Plasmablast | hs00229373_m1 |
| IGJ | Immunoglobulin J Chain | 4.11 | Plasmablast | hs00376160_m1 |
| HP | Haptoglobin | 5.15 | Neutrophil | hs00978377_m1 |
| TCN1 | Transcobalamin 1 | 5.15 | Neutrophil | hs01055542_m1 |
| ELA2 | Neutrophil elastase | 5.15 | Neutrophil | hs00236952_m1 |
| OLR1 | Oxidized Low Density Lipoprotein Receptor 1 | 5.15 | Neutrophil | hs00234028_m1 |
| CEACAM6 | CEA Cell Adhesion Molecule 6 | 5.15 | Neutrophil | hs00366002_m1 |
| AZU1 | Azurocidin 1 | 5.15 | Neutrophil | hs01106962_m1 |
| ARG1 | Arginase 1 | 5.15 | Neutrophil | hs00968978_m1 |
| CEACAM8 | CEA Cell Adhesion Molecule 8 | 5.15 | Neutrophil | hs00266198_m1 |
| CAMP | Cathelicidin Antimicrobial Peptide | 5.15 | Neutrophil | hs00189038_m1 |
| MMP8 | Matrix metalloproteinase-8 | 5.15 | Neutrophil | hs01029060_m1 |
| MPO | Myeloperoxidase | 5.15 | Neutrophil | hs00924296_m1 |
| CKAP4 | Cytoskeleton Associated Protein 4 | 3.2 | Myeloid lineage | hs00199135_m1 |
| DEFA3 | Defensin Alpha 3 | 5.15 | Neutrophil | hs00414018_m1 |
| DEFA1 | Defensin Alpha 1 | 5.15 | Neutrophil | hs00234383_m1 |
| LTF | Lactotransferrin | 5.15 | Neutrophil | hs00914330_m1 |
| DEFA4 | Defensin Alpha 4 | 5.15 | Neutrophil | hs010566650_m1 |
| LY96 | Lymphocyte Antigen 96 | 3.2 | Myeloid lineage | hs01026734_m1 |
| BST1 | Bone Marrow Stromal Cell Antigen 1 | 3.2 | Myeloid lineage | hs01070189_m1 |
| BCL6 | B-Cell Lymphoma 6 | 3.2 | Myeloid lineage | hs01115889_m1 |
| ARHGAP9 | Rho GTPase Activating Protein 9 | 5.7 | Myeloid Lineage | hs01037142_m1 |
| MKNK1 | MAPK Interacting Serine/Threonine Kinase 1 | 3.2 | Myeloid lineage | hs00374375_m1 |
| NCF4 | Neutrophil Cytosolic Factor 4 | 3.2 | Myeloid lineage | hs01055674_m1 |
| HCK | HCK Proto-Oncogene, Src Family Tyrosine Kinase | 3.2 | Myeloid lineage | hs01067412_m1 |
| ITGAM | Integrin Subunit Alpha M | 3.2 | Myeloid lineage | hs01064804_m1 |
| CD63 | CD63 antigen | 3.2 | Myeloid lineage | hs00156390_m1 |
| IL17R(IL17RA) | Interleukin 17A receptor | 3.2 | Myeloid lineage | hs00234888_m1 |
| GPR97 | Adhesion G Protein-Coupled Receptor G3 | 3.2 | Myeloid lineage | hs00416888_m1 |
| CD55 | CD55 molecule | 3.2 | Myeloid lineage | hs00892618_m1 |
| ANXA3 | Annexin A3 | 4.2 | Inflammation | hs00971411_m1 |
| CR1 | Complement C3b/C4b Receptor 1 | 4.2 | Inflammation | hs01079080_m1 |
| PBEF1 | Pre-B-Cell Colony-Enhancing Factor 1 | 3.2 | Myeloid lineage | hs00237184_m1 |
| H3F3B | H3.3 Histone B | 5.7 | Myeloid Lineage | hs00855159_g1 |
| HMGB2 | High Mobility Group Box 2 | 3.2 | Myeloid lineage | hs01128615_m1 |
| PIK3CD | Phosphatidylinositol-4,5-Bisphosphate 3-Kinase Catalytic Subunit Delta | 5.7 | Myeloid Lineage | hs00192399_m1 |
| SLPI | Secretory Leukocyte Peptidase Inhibitor | 4.2 | Inflammation | hs00268204_m1 |
| FCAR | Fc Fragment Of IgA Receptor | 4.2 | Inflammation | hs00370197_m1 |
| MAPK14 | Mitogen-Activated Protein Kinase 14 | 4.2 | Inflammation | hs01051152_m1 |
| IL18RAP | Interleukin 18 Receptor Accessory Protein | 4.2 | Inflammation | hs00977702_m1 |
| CLEC4D | C-Type Lectin Domain Family 4 Member D | 4.2 | Inflammation | hs01073581_m1 |
| MCEMP1 | Mast Cell Expressed Membrane Protein 1 | 4.2 | Inflammation | hs00545332_m1 |
| NFIX | Nuclear factor I/X | 2.3 | Erythropoiesis | hs00958850_m1 |
| S100A12 | S100 Calcium Binding Protein A12 | 4.2 | Inflammation | hs00194525_m1 |
| S100P | S100 Calcium Binding Protein P | 4.2 | Inflammation | hs00195584_m1 |
| MMP9 | Matrix Metalloproteinase 9 | 4.2 | Inflammation | hs00957562_m1 |
| SERPINA1 | Serpin Family A Member 1 | 4.2 | Inflammation | hs00165475_m1 |
| PGLYRP1 | Peptidoglycan Recognition Protein 1 | 4.2 | Inflammation | hs00175475_m1 |
| GUK1 | Guanylate Kinase 1 | 3.1 | Erythropoiesis | hs00176133_m1 |
| MAP2K3 | Mitogen-Activated Protein Kinase Kinase 3 | 3.1 | Erythropoiesis | hs03005115_m1 |
| ADIPOR1 | Adiponectin Receptor 1 | 3.1 | Erythropoiesis | hs00360422_m1 |
| FLCN | Folliculin | 3.1 | Erythropoiesis | hs00376065_m1 |
| IGF2BP2 | Insulin Like Growth Factor 2 MRNA Binding Protein 2 | 3.1 | Erythropoiesis | hs01118006_m1 |
| PLEK2 | Pleckstrin 2 | 2.3 | Erythropoiesis | hs01026898_m1 |
| PIP5K2A | Phosphatidylinositol-4-Phosphate 5-Kinase Type II Alpha | 2.3 | Erythropoiesis | hs01124167_m1 |
| MXI1 | MAX Interactor 1, Dimerization Protein | 2.3 | Erythropoiesis | hs00365651_m1 |
| BCL2L1 | BCL2 Like 1 | 2.3 | Erythropoiesis | hs04408449_m1 |
| GSPT1 | G1 To S Phase Transition 1 | 3.1 | Erythropoiesis | hs01093019_m1 |
| PPIA | Peptidylprolyl Isomerase A | - | Reference | hs99999904_m1 |


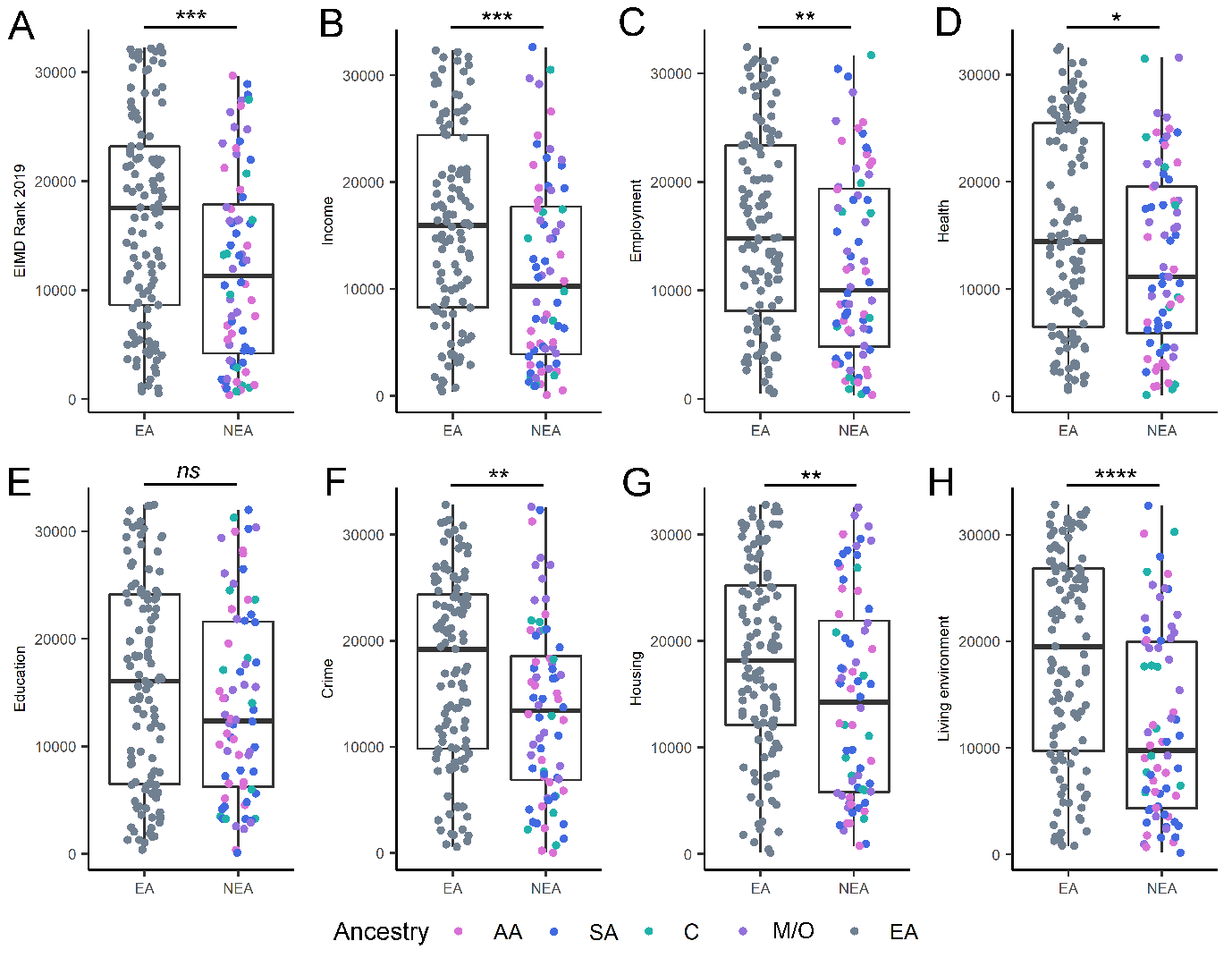


## Figure S2. BILAG-BR patients of non-European ancestry reside in areas of greater relative deprivation.

Boxplot series with overlay jitter points color coded by ancestral group, show ranked deprivation indices from 1 (most deprived area) to 32,844 (least deprived area). Compared with patients of European ancestry (EA), patients of non-European ancestry (NEA) reside in areas of significantly higher overall deprivation as measured by the 2019 English Index of Multiple Deprivation (EIMD, **A**). Composite domains of deprivation Income (**B**), Employment (**C**), Health (**D**), Education (**E**), Crime (**F**), Barriers to Housing and Services (**G**) and Living Environment (**H**) show NEA patients reside in areas of significantly higher relative deprivation across all domains with the exception of Education (**E)**. * p ≤ 0.05, ** p ≤ 0.01, *** p ≤ 0.001, **** p ≤ 0.0001, *ns* non significant. AA, African ancestry; SA, Subcontinental Asian; C, Chinese and other Asian; O/M Other or Mixed heritage; EA, European ancestry.

| **Table S2. Baseline characteristics of BILAG-BR patients of European and non-European ancestry.** | | | | |
| --- | --- | --- | --- | --- |
| **Clinical characteristics** | **Overall**  **n = 213** | **European Ancestry**  **n = 128** | **Non-European Ancestry**  **n = 85** | **p value** |
| **Female patient, (%)** | 196 (92) | 121 (94%) | 75 (88.2) | 0.09 |
| **Age (years), median (IQR)** | 39 (30, 50) | 41 (34, 52) | 36 (26, 47) | **0.001** |
| **Disease duration (years), median (IQR)** | 11 (7, 18) | 12 (7, 18) | 11 (7, 16) | 0.569 |
| **Current smoker (n/N, %)** | 47/142 (33) | 37/85 (44) | 10/52 (19) | **0.001** |
| **Index of multiple deprivation (Rank), median (IQR)** | 14100 (5993, 21990) | 17526 (8633, 23176) | 11311 (4197, 17847) | **0.002** |
| **BILAG-BR registration therapy**  Belimumab  Mycophenolate mofetil  Rituximab | 19 (9)  32 (15)  162 (76) | 12 (9)  16 (13)  100 (78) | 7 (8)  16 (19)  62 (73) | 0.549 |
| **BILAG A or B score, n (%)**  Constitutional  Mucocutaneous  Neuropsychiatric  Musculoskeletal  Cardiorespiratory  Gastroenterology  Ophthalmic  Renal  Haematology | 19 (9.0)  98 (46.0)  26 (12.2)  88 (41.3)  32 (15.0)  7 (3.2)  11 (5.1)  79 (37.1)  8 (3.7) | 12 (9.4)  67 (52.3)  14 (10.9)  55 (42.9)  20 (15.6)  3 (2.3)  7 (5.4)  42 (32.8)  4 (3) | 7 (8.2)  31 (24.2)  12 (14.1)  33 (38.8)  12 (14.1)  4 (4.7)  4 (4.7)  37 (44)  4 (4.7) | **0.029**  0.644  0.078 |
| **BILAG numerical score, median (IQR)** | 18 (12, 24) | 17 (13, 25) | 19 (12, 24) | 0.642 |
| **SLEDAI Score, median (IQR)** | 8 (4, 13) | 8 (4, 12) | 8 (4, 14) | 0.274 |
| **SLICC damage index, median (IQR)** | 0 (0,1) | 0 (0,1) | 0 (0, 1) | 0.695 |
| **Full blood count, median (IQR)**  Hb (g/L)  WCC (x10^9^ / L)  Neutrophils (x10^9^ / L)  Lymphocytes (x10^9^ / L)  Platelets (x10^9^ / L) | 121.0 (108.0, 133.0)  6.5 (4.5, 9.0)  4.6 (2.9, 7.0)  1.0 (0.7, 1.6)  261 (201, 331) | 124.0 (115.0, 134.5)  6.5 (4.8, 8.9)  4.6 (2.9, 6.7)  1.1 (0.7, 1.6)  268 (205, 345) | 115.5 (102.2, 127.0)  6.5 (4.0, 9.3)  4.7 (2.9,7.5)  1.0 (0.7, 1.5)  253 (200, 307) | **0.004**  0.976  0.866  0.311  0.080 |
| **Total IgG (g/L), median (IQR)** | 12.9 (9.5, 16.7) | 10.9 (8.5, 14.25) | 16.2 (12.5, 20.9) | **<0.000** |
| **Low C3 or C4, n (%)** | 103 (48) | 55 (43.0) | 48 (56.5) | **0.045** |
| **Concurrent Immunosuppressant, n (%)**  Any agent (MMF, MTX, CNI, AZA)  Mycophenolate mofetil | 104 (49)  77 (36) | 65 (50.7)  45 (35.2) | 39 (45.8)  32 (37.6%) | 0.483  0.711 |
| **Anti-malarial, n (%)** | 109 (51) | 65 (50.7) | 44 (51.8) | 0.888 |
| **Oral glucocorticoid dose (mg), median (IQR)** | 10 (7.5, 20) | 10 (5, 20) | 10 (7.5, 20) | 0.180 |
| **Immunoprecipitation and ELISA** | **n = 158** | **n = 96** | **n = 62** |  |
| U1RNP-Sm positive, n (%)  Ro-60, n (%)  La,  Ro-52 ELISA, n (%)  dsDNA ELISA n (%)  Cardiolipin ELISA n (%) | 42 (27)  56 (35)  11 (7)  30 (19)  86 (54)  20 (13) | 11 (11.5)  28 (29.2)  7 (7.3)  16 (16.7)  44 (45.8)  12 (12.5) | 31 (50.0)  28 (45.1)  4 (6.5)  14 (22.6)  42 (67.7)  8 (12.9) | **<0.000**  **0.040**  0.839  0.354  **0.006**  0.960 |
| **Response to rituximab 6 months** | **n = 110** | **n = 65** | **n = 45** |  |
| BILAG responder (complete or partial), n (%) | 70 (63) | 45 (69) | 25 (56) | 0.142 |


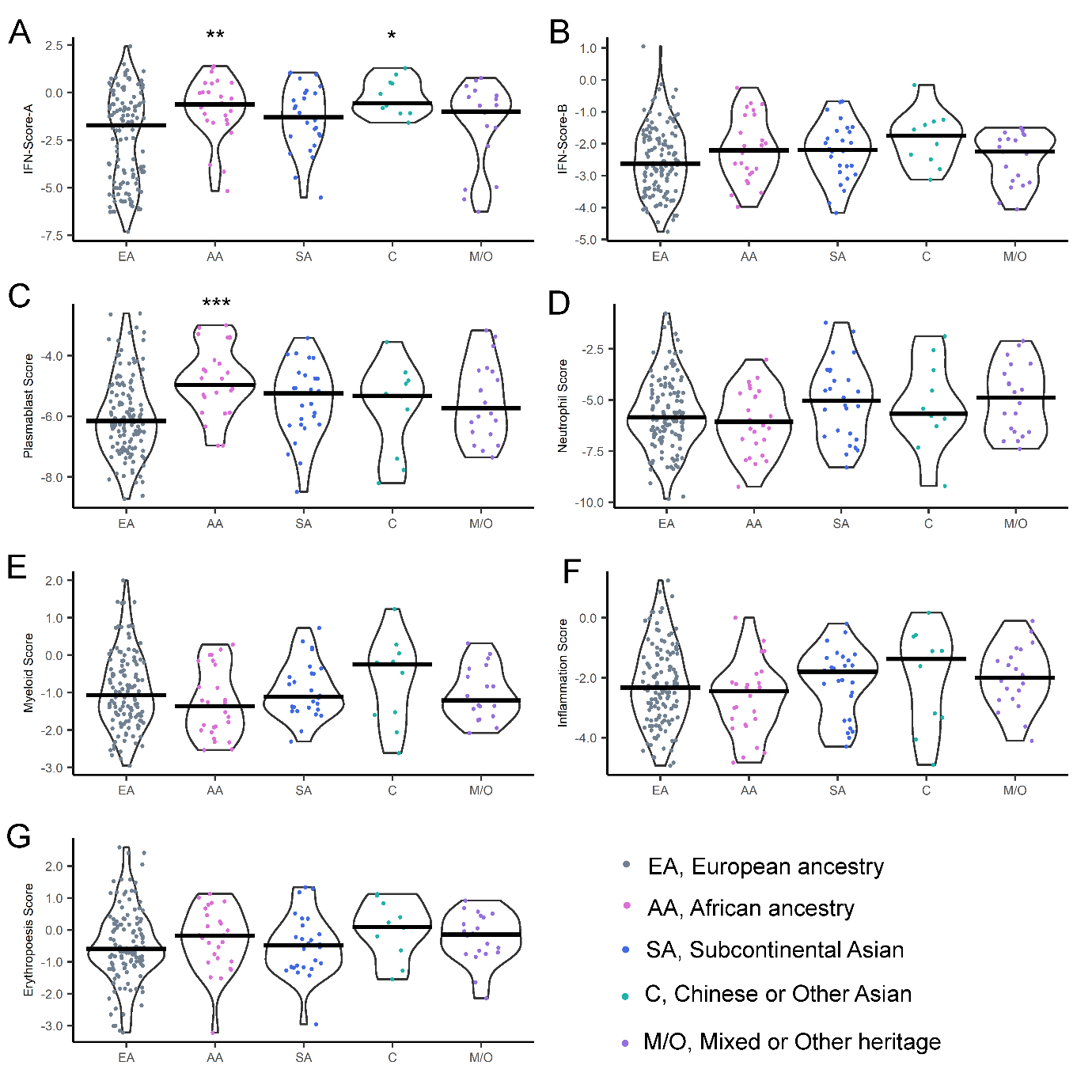


## Figure S3. Interferon and Plasmablast annotated gene expression scores vary by ancestral background.

Violin plot series with overlay jitter points color coded by ancestral group, show distribution and median (horizontal bar) expression of IFN-Score-A (**A**) which comprises interferon stimulated genes (ISGs) typical of the global type-I IFN signature, IFN-Score-B (**B**) comprising more diversely regulated ISGs, Plasmablast (**C**), Neutrophil (**D**), Myeloid (**E**), Inflammation (**F**) and Eyrthropoesis (**G**) -annotated gene expression scores. Consistent with existing literature, patients of European ancestry show lower IFN-Score-A expression, but also display a bimodal distribution of IFN-Score-A which is not observed in patients of non-European ancestries. Significantly higher Plasmablast Score is evident among patients of African Ancestry. * p ≤ 0.05, ** p ≤ 0.01 for comparison against European ancestry (EA) group by Kruskal Wallace test and post-hoc pairwise Dunn’s test.


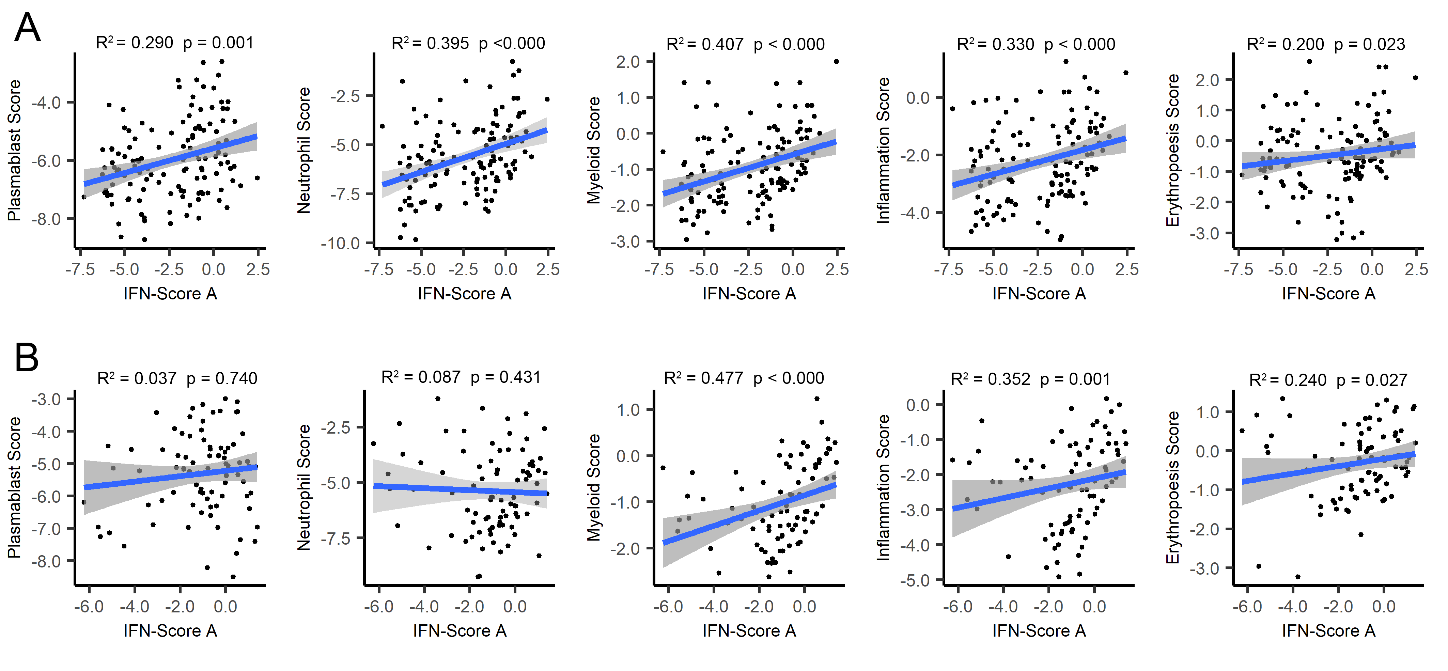


## Figure S4. Relationship between IFN-Score-A and other functionally annotated gene expression scores varies by patient ancestry.

Scatterplot series show relationship between Plasmablast, Neutrophil, Myeloid lineage, Inflammation and Eyrthropoesis annotated expression scores and interferon pathway activation as measured by IFN-Score-A in patients of European (**A**) and non-European ancestry (**B**). Regression line shown in blue and standard error in grey. All gene expression scores are shown as ΔCt from reference gene PPIA reflected across zero such that higher values indicate higher expression.


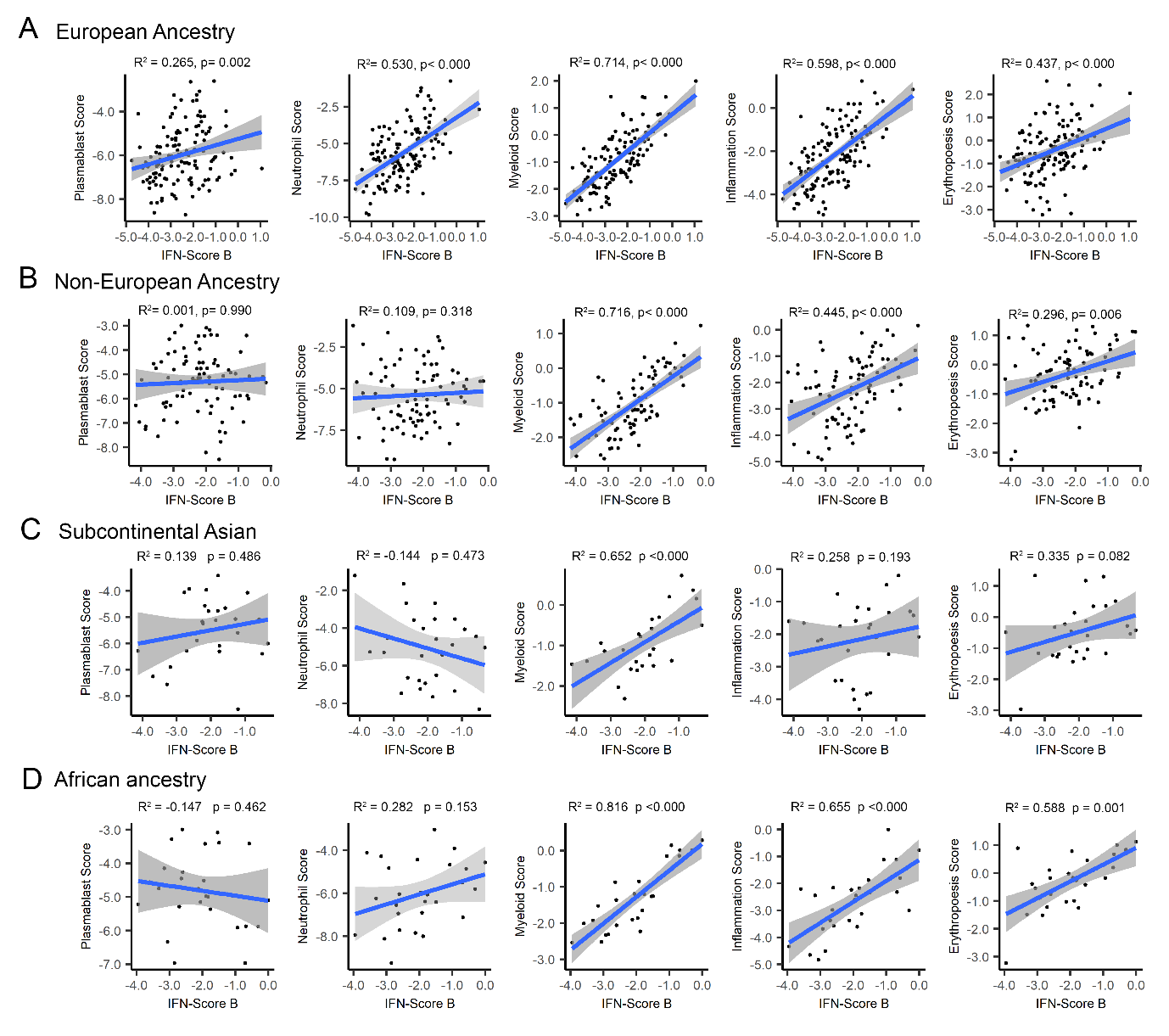


## Figure S5. Commonalities in the relationship between IFN-Score-B and other functionally annotated gene expression scores varies displays are shared between Subcontinental Asian and African ancestry patients.

Scatterplot series show relationship between Plasmablast, Neutrophil, Myeloid lineage, Inflammation and Eyrthropoesis annotated expression scores and interferon pathway activation as measured by IFN-Score-B in patients of European (**A**) ancestry, non-European ancestry collectively (**B**) and separately the two larger composite non-European ancestry populations; Subcontinental Asian (**C**) and African ancestry (**D**) patients. The dissociation between IFN-Score-B and Plasmablast and Neutrophil- annotated scores observed collectively in non-European ancestry patients is apparent in both Subcontinental Asian and African ancestry patients independently. Regression line shown in blue and standard error in grey. All gene expression scores are shown as ΔCt from reference gene PPIA reflected across zero such that higher values indicate higher expression.

| **Table S3: Summary of similarities and distinguishing features between gene expression clusters** | | | | | | | |
| --- | --- | --- | --- | --- | --- | --- | --- |
|  | **Clinical characteristics** | **NEA-3**  **All signatures high** | **EA-1**  **All signatures high** | **NEA-2**  **IFN-high, Neutrophil-myeloid-inflammation low** | **EA-2**  **IFN-high, Neutrophil-myeloid-inflammation-erythropoesis low** | **NEA-1**  **IFN low, Neutrophil-myeloid- inflammation high** | **EA-3**  **All signatures low** |
| **Ancestry** | **European ancestry** | - | + | - | + | - | + |
|  | **Non-European ancestry** | + | - | + | - | + | - |
| **Gene expression signature** | **IFN** | + | + | + | + | - | - |
|  | **Neutrophil-Myeloid-Inflammation** | + | + | - | - | + | - |
|  | **Erythropoesis** | + | + | + | - | + | - |
| **Organ domain involvement** | **Mucocutaneous (%)** | 36 | 58 | 43 | 67 | 22 | 44 |
|  | **Renal (%)** | 44 | 61 | 48 | 21 | 67 | 16 |
| **Seropositivity** | **RNP** | 63 | 10 | 50 | 33 | 0 | 0 |
|  | **dsDNA** | 79 | 56 | 63 | 52 | 50 | 23 |
| **Response to RTX** | **BILAG response 6 months** | 85 | 61 | 41 | 72 | 13 | 81 |


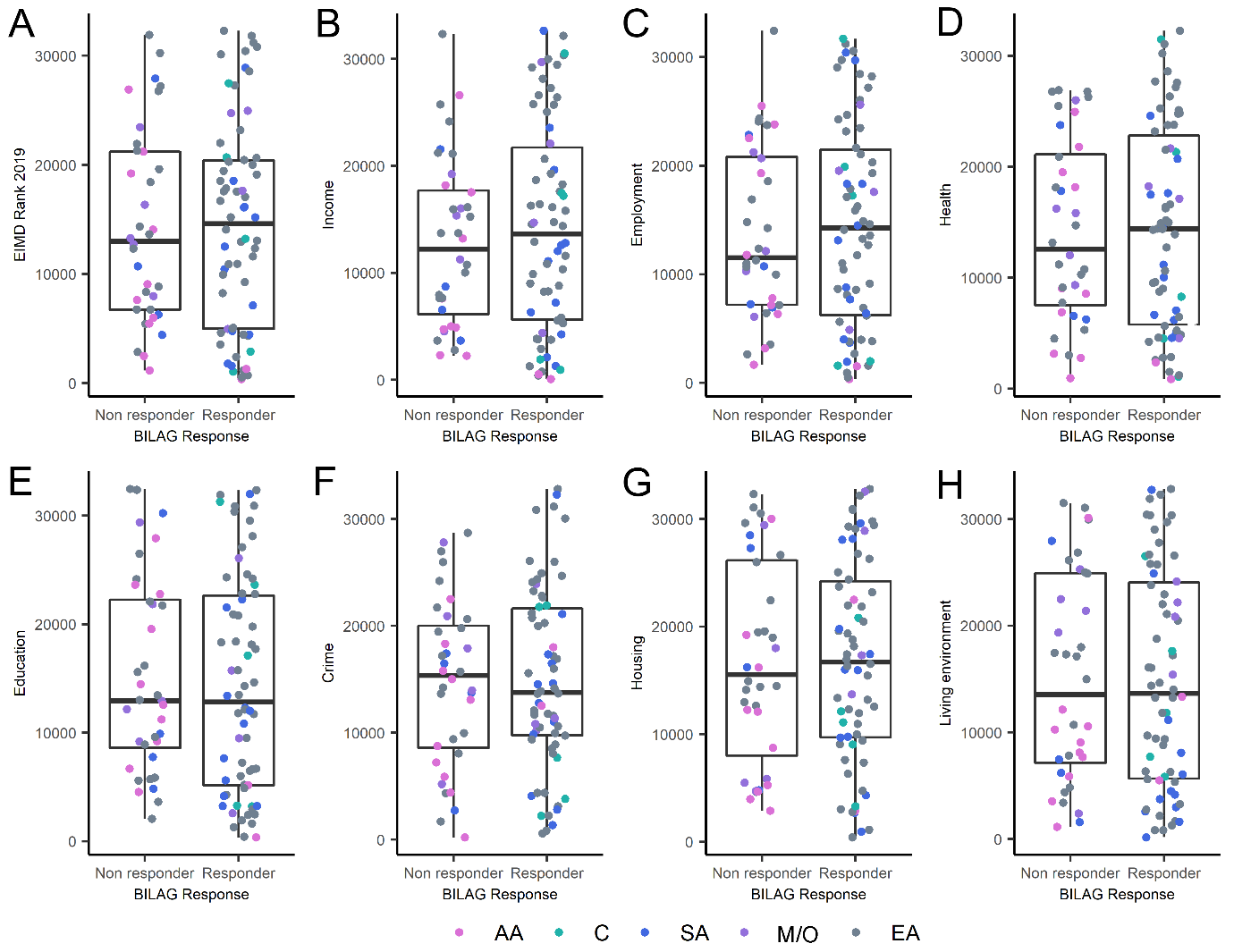


## Figure S6. BILAG response following rituximab therapy is not associated with indices of socioeconomic deprivation.

Boxplot series with overlay jitter points color coded by ancestral group, show ranked deprivation indices from 1 (most deprived area) to 32,844 (least deprived area). Overall deprivation as measured by the 2019 English Index of Multiple Deprivation (EIMD, **A**) and composite domains of deprivation Income (**B**), Employment (**C**), Health (**D**), Education (**E**), Crime (**F**), Barriers to Housing and Services (**G**) and Living Environment (**H**) did not significantly differ between patients achieving a BILAG response to rituximab at 6 months (Responders) and patients not achieving response (Non Responders).

| **Table S4: Characteristics of BILAG responders and non-responders following cycle 1 rituximab** | | | | |
| --- | --- | --- | --- | --- |
|  | **European Ancestry** | | **Non-European Ancestry** | |
| **Clinical characteristics** | **Responders**  **n = 45** | **Non-Responders**  **n = 20** | **Responders**  **n = 25** | **Non-Responders**  **n = 20** |
| **Female patient, (%)** | 43 (95) | 16 (80) | 20 (80) | 18 (90) |
| **Age (years), median (IQR)** | 41 (33, 52) | 42 (38, 58) | 38 (26, 44) | 38 (25, 50) |
| **Disease duration (yrs), median (IQR)** | 13, (8,21) | 10 (5, 18) | 12 (7, 16) | 12 (8, 20) |
| **Current smoker, n/N (%)** | 14/30 (47) | 8/15 (53) | 1/16 (6) | 4/15 (27) |
| **Index of multiple deprivation (Rank), median (IQR)** | 16679 (8756, 20,535) | 14353 (8746, 20535) | 12518 (3650, 18075) | 10699 (6126, 17769)  0.484 |
| **BILAG A or B score, n (%)**  Constitutional  Mucocutaneous  Neuropsychiatric  Musculoskeletal  Cardiorespiratory  Gastroenterology  Ophthalmic  Renal  Haematology | 5 (11)  27 (60)  6 (13)  24 (53)  7 (16)  3 (7)  1 (2)  19 (42)  1 (2) | 5 (25)  13 (65)  6 (30)  11 (55)  4 (20)  0 (0)  2 (10)  8 (40)  1 (6) | 9 (36)  11 (44)  1 (4)  9 (36)  4 (16)  3 (12)  1 (4)  13 (52)  0 (0) | 2 (10)  7 (35)  5 (25)  9 (45)  4 (20)  1 (5)  1 (5)  11 (55)  4 (20) |
| **Numerical BILAG, median (IQR)** | 21 (16, 25) | 22 (20, 28) | 15 (13,22) | 21 (20, 30) |
| **SLEDAI Score, median (IQR)** | 10 (8, 14) | 8 (7, 11) | 8 (4, 14) | 8 (6,13) |
| **SLICC damage index, median (IQR)** | 0 (0, 2) | 1 (0, 4) | 0 (0,1) | 1 ( 0, 2) |
| **Total IgG (g/L), median (IQR)** | 10.6 (8.4, 13.9) | 11.7 (9.5, 15.2) | 12.7 (10.6, 18.0) | 15.0 (9.5, 16.5) |
| **Low C3 or C4, n (%)** | 23 (51) | 7 (35) | 14 (56) | 12 (60) |
| **Concurrent Immunosuppression n (%)**  Any agent (MMF, MTX, CNI, AZA)  Mycophenolate mofetil | 22 (48)  15 (33) | 8 (40)  6 (30) | 10 (40)  8 (32) | 5 (25)  4 (20) |
| **Anti-malarial, n (%)** | 26 (58) | 10 (50) | 14 (56) | 11 (55) |
| **Oral glucocorticoid dose at baseline (mg), median (IQR)** | 10 (7, 18) | 10 (5, 20) | 10 (8, 10) | 15 (10,20) |
| **Oral glucocorticoid dose at 6 months (mg), median (IQR)** | 5 (0, 10) | 5 (0, 10) | 5 (3, 9) | 10 (6, 13) |
| **Change in oral glucocorticoid dose at 6 months (mg), median (IQR)** | -1 (-13, 0) | 0 (-5, 0) | -4 (-10, 0) | 0 (-9, +2) |
| **Immunoprecipitation and ELISA** | **n = 37** | **n = 19** | **n = 20** | **n = 13** |
| U1RNP-Sm positive, n (%)  Ro-60 n (%)  La  Ro-52 ELISA n (%)  dsDNA ELISA n (%)  Cardiolipin ELISA n (%) | 7 (19)  12 (32)  2 (5)  6 (16)  20 (54)  3 (8) | 2 (11)  4 (21)  1 (5)  2 (10)  9 (47)  3 (15) | 9 (45)  8 (32)  0 (0)  2 (10)  11 (55)  1 (5) | 5 (38)  5 (38)  1 (7)  2 (15)  9 (69)  3 (23) |

| **Table S5. Response to rituximab by 2011 UK Census ancestral group** | |
| --- | --- |
| **Ancestral background**  **UK 2011 Census category** | **BILAG response**  **6 months post RTX** |
| **Black, African, Caribbean or Black British, n/N (%)** Black African  Black Caribbean  Other Black background | **3/14 (21)**  1/7  2/6  0/1 |
| **Asian or British Asian, n/N (%)** Indian  Pakistani  Chinese  Other Asian background | **18/22 (82)**  6/9  6/7  3/3  3/3 |
| **Mixed** **or multiple ethnic groups, n/N (%)** White and Black Caribbean  White and Black African Any other Mixed or Multiple ethnic background | **3/5 (60)**  2/3  0/1  1/1 |
| **White, n/N (%)** English, Welsh, Scottish, Northern Irish or British  Irish  Any other White background | **45/65 (69)**  42/62  2/2  1/1 |
| **Other, n/N (%)** | **1/4 (25)** |

| **Table S6: Differential response to rituximab between non-European ancestry gene expression clusters itemized by patient ancestry** | | | | |
| --- | --- | --- | --- | --- |
| **Response to rituximab 6 months** | **All patients** | **NEA Cluster 1**  **n = 8**  **IFN low,**  **Neutrophil-myeloid- inflammation high** | **NEA Cluster 2**  **n = 17**  **IFN high,**  **Neutrophil-myeloid- inflammation low** | **NEA Cluster 3**  **n = 20**  **All signatures high** |
| **BILAG responder (complete or partial), n (%)**  African Ancestry  South Asian  Other Asian (incl. Chinese)  Other (inc. Mixed) | **25/45 (56)**  3/14 (21)  12/16 (75)  6/6 (100)  4/9 (44) | **1 (12.5)**  0/1 (0)  0/4 (0)  -  1/3 (33) | **7 (41.2)**  2/10 (20)  5/5 (100)  -  0/2 (0) | **17 (85.0)**  1/3 (33)  7/7 (100)  6/6 (100)  3/4 (75) |

## Appendix 2: Contributors to the MASTERPLANS Consortium

The University of Manchester: Prof Katherine Payne; Dr Mark Lunt; Prof Niels Peek; Dr Nophar Geifman; Dr Sean Gavan; Dr Gillian Armitt; Dr Patrick Doherty; Dr Jennifer Prattley; Dr Narges Azadbakht; Angela Papazian; Dr Helen Le Sueur; Carmen Farrelly; Clare Richardson; Zunnaira Shabbir; Lauren Hewitt; Dr Emily Sutton; Alison Fountain, Patrick Doherty.

University of Bath: Prof Neil McHugh.

University of Birmingham: Prof Caroline Gordon; Prof Stephen Young.

University of Cambridge: Prof David Jayne; Prof Vern Farewell; Dr Li Su.

Imperial College London: Prof Matthew Pickering; Prof Elizabeth Lightstone; Dr Alyssa Gilmore; Prof Marina Botto.

King's College London: Prof Timothy Vyse; Dr David Lester Morris; Prof David D’Cruz.

University of Liverpool: Prof Michael Beresford; Prof Christian Hedrich; Dr Angela Midgley; Dr Jenna Gritzfeld.

University College London: Prof Michael Ehrenstein; Prof David Isenberg; Mariea Parvaz.

MASTERPLANS Patient and Public Involvement Group: Jane Dunnage; Jane Batchelor Elaine Holland; Pauline Upsal
